# Emerging trends in direct air capture of CO_2_: a review of technology options targeting net-zero emissions

**DOI:** 10.1039/d2ra07940b

**Published:** 2023-02-15

**Authors:** Yasser Abdullatif, Ahmed Sodiq, Namra Mir, Yusuf Bicer, Tareq Al-Ansari, Muftah H. El-Naas, Abdulkarem I. Amhamed

**Affiliations:** a College of Science and Engineering, Hamad Bin Khalifa University, Qatar Foundation Education City Doha Qatar; b Qatar Environment and Energy Institute (QEERI) Doha Qatar aamhamed@hbku.edu.qa; c Gas Processing Center (GPC), Qatar University Doha Qatar

## Abstract

The increasing concentration of carbon dioxide (CO_2_) in the atmosphere has compelled researchers and policymakers to seek urgent solutions to address the current global climate change challenges. In order to keep the global mean temperature at approximately 1.5 °C above the preindustrial era, the world needs increased deployment of negative emission technologies. Among all the negative emissions technologies reported, direct air capture (DAC) is positioned to deliver the needed CO_2_ removal in the atmosphere. DAC technology is independent of the emissions origin, and the capture machine can be located close to the storage or utilization sites or in a location where renewable energy is abundant or where the price of energy is low-cost. Notwithstanding these inherent qualities, DAC technology still has a few drawbacks that need to be addressed before the technology can be widely deployed. As a result, this review focuses on emerging trends in direct air capture (DAC) of CO_2_, the main drivers of DAC systems, and the required development for commercialization. The main findings point to undeniable facts that DAC's overall system energy requirement is high, and it is the main bottleneck in DAC commercialization.

## Abstract 2

The increasing concentration of carbon dioxide (CO_2_) in the atmosphere has compelled researchers and policymakers to seek urgent solutions to address the current global climate change challenges.

## Graphical abstract

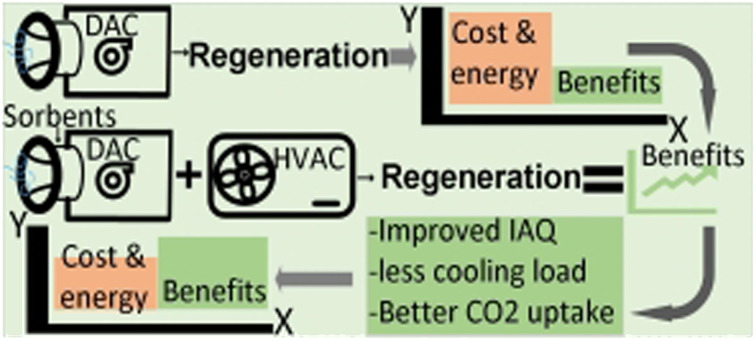

## Introduction

**Fig. 1:**
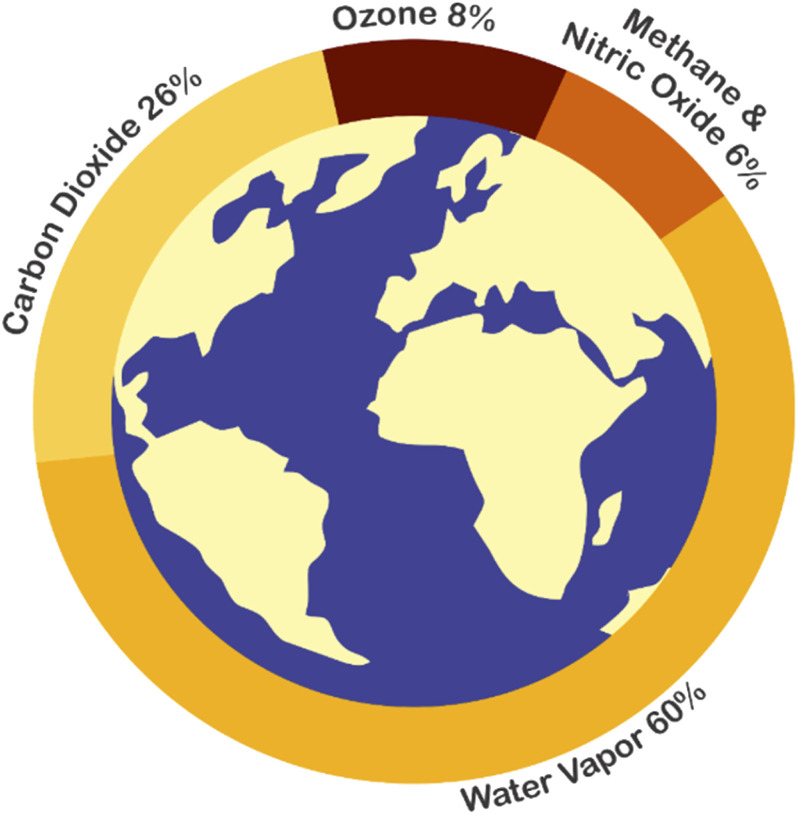
Greenhouse gases percentage in the atmosphere, adapted from ref. 7.

The increase in the Earth's temperature took a new turn in 1950 and has steadily continued through the 21st century. By the year 2020, however, the global mean temperature was 1.02 °C above the preindustrial era.^1,2^ The Paris agreement was signed to maintain the global mean temperature below 2 °C to avoid catastrophic global warming consequences.^3^ By achieving the Paris agreement objective, 9 to 10 billion people will not be exposed to heatwaves, and 85 million people will not be affected by flooded rivers. Moreover, five hundred million people will not suffer from water stress, and 3 million square kilometers will remain viable for agriculture (half of the country like India).^4^ The review papers published by 97% of climate experts and the Intergovernmental Panel on Climate Change (IPCC) reported that the origin of climate change is the high emission rate of Green House Gases (GHGs), mainly CO_2_.^5^ The GHGs cause global warming because these gases can absorb the infrared radiation reflected from the earth and reemit it, thereby raising the earth temperature. GHGs such as methane (CH_4_), halocarbons, nitrous oxide (N_2_O) and ozone (O_3_) exist in the atmosphere with low concentration while the high concentration GHGs are water vapor (H_2_O) and carbon dioxide (CO_2_) as shown in Fig. 1.^6^

The most concentrated GHGs in the atmosphere are water vapor and CO_2_ and are the leading cause of global warming. In fact, water vapor is more concentrated and can absorb more infrared waves than carbon dioxide.^8^ The global temperature increases due to anthropogenic activities, especially when humans use fossil fuels that emit CO_2_ and with higher CO_2_ concentrations, the atmosphere gets hotter and more humid.^9^ Thus, reducing the CO_2_ emissions in the atmosphere will result in low water vapor concentration and reduced global mean temperature.^10,11^ Preventive and remediation methods can reduce CO_2_ emissions. The preventive approach includes using renewable energy systems and improving systems efficiency,^12^ while the remediation includes capturing, storing, and utilizing CO_2_.^13^ Although the whole world exerts efforts to reduce CO_2_ emission by preventive methods, billions of tons of CO_2_ is still being emitted into the atmosphere. Considering the continuous emissions, IPCC proposed CO_2_ capture as a necessary.

**Fig. 2:**
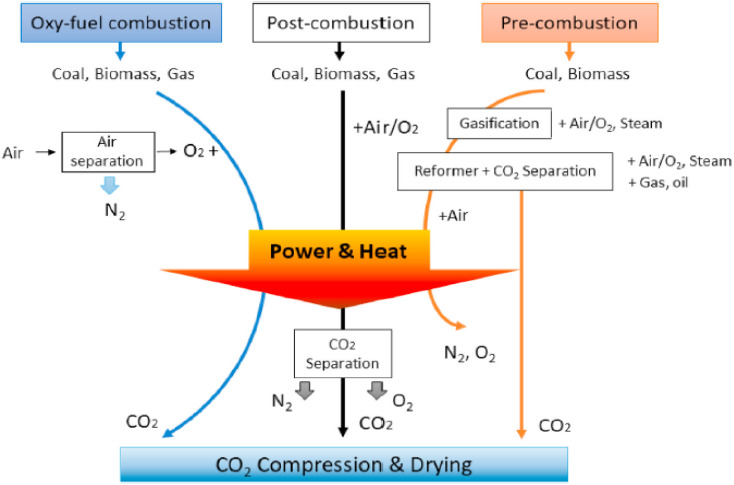
The different carbon capturing systems taken from ref. 16 with permission from Elsevier, copyright 2021.

There are two types of CO_2_ capture technologies: the conventional stationary sources^14^ and the direct air capture (which removes CO_2_ directly from the atmosphere). The conventional CO_2_ capture technology prevents the emitted GHGs from spreading to the atmosphere.^15,16^ As shown in Fig. 2, the conventional CO_2_ capture technique is subdivided technology to limit CO_2_ emissions^17^ into pre-combustion, oxy-fuel combustion, and post-combustion capture.^18,19^ In pre-combustion carbon capture, a production of syngas (hydrogen and carbon monoxide mixture) from fuel reforming is followed by CO_2_ separation process.^20^ The oxy-fuel combustion technology includes fossil fuel burning in the presence of pure oxygen.^21^ The post-combustion approach involves capturing CO_2_ from flue gas (end of pipe treatment approach).^16,22^ The three mentioned conventional carbon capture techniques prevent emissions of CO_2_ to the atmosphere from burning of fossil-based fuels. On the other hand, Negative Emissions Technologies (NETs) including direct air capture technology (DAC) creates an outlet to directly capture the existing CO_2_ from the atmosphere to achieve negative emission goals.^23^ Although the IPCC proclaimed that fossil fuel usage was needed to be immediately reduced in 1990,^24^ fossil fuel global primary energy consumption represented 84.3% in 2019.^25^ As a result, the latest report from IPCC stated that the contribution of NETs are required to stabilize the CO_2_ emission at double the preindustrial levels by the second half of 21st century.^26^ Moreover, the need for rapid deployment of negative carbon technologies were proposed by Paris talks and Nation Research Council (NRC) to meet the 2 °C limit and net-zero carbon in the later part of 21st century goals.^27,28^

**Fig. 3:**
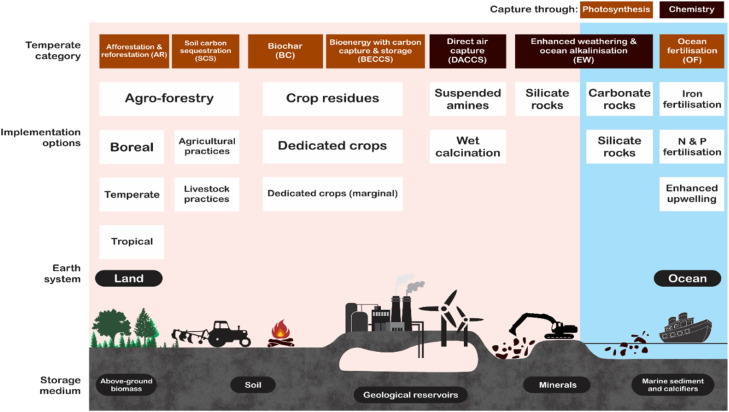
Infographic representations of NETs (adapted from ref. 41 licensed under CC BY 3.0).

Negative emissions technologies (NETs) are carbon dioxide removal (CDR) techniques from the atmosphere, such as direct air capture (when carbon removal is achieved through physicochemical processes such as adsorption, absorption, ocean alkalinity enhancement and soil mineralization) or indirect air capture (when carbon removal is achieved through biological processes such as bioenergy with carbon capture and storage (BECCS), ocean fertilization, afforestation, biochar and algae culture).^29,30^ Under direct air capture, there is adsorption, which is the capture of CO_2_ in the pores of solid sorbents (physisorption)^31,32^ or the reaction of acidic natured CO_2_ with basic sites on solid materials (chemisorption).^32,33^ Alternatively, CO_2_ can be captured in the liquid volume in the process known as absorption. In absorption, reactive compounds (such as NaOH, Ca(OH)_2_, KOH and amines) are dissolved in liquid phase, which allows CO_2_ from air to be retained within the liquid volume.^34^ Ocean alkalinity enhancement is an absorption process in which CO_2_ is absorbed in ocean water and reacts with ocean minerals to neutralize ocean acidification.^35,36^ This process alone captures about one-third of the global CO_2_ emissions from the atmosphere.^34^ Soil mineralization is another direct air capture approach, it is the largest land-based carbon sink on the planet and it is capable of capturing about 3 GtC year^−1^.^37^ Soil carbon mineralization follows three approaches,^38^ (i) buildup of organic carbon as a result of plant growth; (ii) rock weathering (helping the breakdown of inorganic carbon in soil solution); and (iii) precipitation of carbonate materials. However, ocean alkalinity enhancement and soil mineralization were rarely investigated in the literature and more studies are required to show if the technology can be used at scale.^39^ Detailed pictorial representations of NETs are shown in Fig. 3 below. BECCS is the most researched NETs under indirect air capture.^40^

CO_2_ from the atmosphere is captured naturally by microorganisms and plants through photosynthesis, thereby producing biomass. Since biomass is considered a clean source of energy, the biomass obtained can be used to generate electricity *via* thermoelectric power plant, thereby translating to negative emissions,^34,42^ however, its use is dependent on the availability of biomass, land and storage.^43^ Afforestation is another indirect air capture that has the capacity to store large amount of CO_2_. Just like soil mineralization, afforestation is a technique that can be deployed anytime because it is readily available.^44^ Planting new trees or better management of the existing forests can increase the natural rates of carbon capture from the atmosphere,^45^ although it has been reported in the literature that this may lead to loss of biodiversity,^46^ loss of valuable land for crops,^47^ and increase in loss of ice and local warming in high altitude forest.^48^ Ocean fertilization increases biomass productivity on the ocean floor when micronutrients (such as iron) and macronutrients (such as phosphorus and nitrogen) are dissolved into the ocean, whereby the expected microorganism sinks to the ocean floor.^49^ Algae (microalgae and seaweeds) culture has been reported to have photosynthetic efficiency that is ten times higher than plants.^50^ Microalgae culture has the capacity to capture CO_2_ from different sources, such as from flue gas point sources and distributed sources like the atmosphere.^50^ The most remarkable part of CO_2_ biosequestration procedure is the recycling of part of biomass in the form of biochar. Biochar is produced through pyrolysis or gasification of biomass.^51^ Currently, biomass remains are burned or decomposed in soil; and as a result, large amount of the CO_2_ captured through photosynthesis is emitted to the atmosphere. When biomass is converted to biochar for soil nourishment, CO_2_ emission may be reduced by as much as 1.8 Gt year^−1^ without CCS.^52^

About half of CO_2_ yearly emissions are from distributed sources. The fact that DAC systems can remove CO_2_ from both distributed and point sources, are not location specific, do not have contamination issues such as (NO_*x*_ and SO_*x*_), and has small footprint, shows the necessity of deploying DAC over point sources and other NETs.^53^ However, capturing CO_2_ from dilute air is an energy intensive process. The minimum CO_2_ separation energy required in case of dilute air (400 ppmCO_2_) was calculated to be about 20 kJ per molCO_2_,^16,29–31^ while the minimum energy required to capture from flue gas using benchmark aqueous MEA is 8.4 kJ per molCO_2_.^54^ DAC is a relatively new technology that is still in its early commercial stages. The early startups that have contributed immensely to DAC commercialization are Carbon Engineering in Canada, Climeworks in Switzerland and Global Thermostat in the United States.^55^ Different DAC studies have shown that chemisorption is more relevant to DAC than physisorption (activated carbon, zeolites, and metal–organic frameworks) because physisorption has poor performance in capturing CO_2_ at low concentration streams in the presence of water vapor.^56,57^

The high energy requirements of DAC lead to high capital and operating costs, which is a major challenge of the existing technologies.^58^ The costs are reported in the literature for three main types of DAC systems, which are high temperature aqueous solutions,^59–64^ low temperature solid sorbents,^65-67^ and moisture swing solid sorbents.^68^ However, these reported costs were not comparable due to different assumptions and outputs. Fasihi *et al.*^69^ recalculated these costs based on fixed assumption and reported cost range of 115-388, 120-244 and 99 EUR per ton CO_2_ for high temperature aqueous solutions, low temperature solid sorbents and moisture swing solid sorbents, respectively. A DAC cost of Gigaton scale at less than $100 per ton CO_2_ by 2050 is needed for the technology to achieve great climate impact, which highlights the need for cost reduction in DAC technologies.^70^ Many studies have been carried out to estimate the potential cost of DAC to show if it would be a climate change valuable solution in the future. Simon *et al.*^71^ examined a generic DAC technology, and claimed that capturing CO_2_ at 220 EUR per ton CO_2_ is currently possible but indicated that the calculated cost could be provided by more research into kinetics and capturing thermodynamics. Moreover, the authors estimated a range of cost from 75 to 800 EUR per ton CO_2_ based on pessimistic and optimistic scenarios. House *et al.*^72^ pointed out that the literature underestimated the cost of DAC, and the current cost of DAC is as high as 750 EUR per ton CO_2_, however, the cost has the potential to reach 225 EUR per ton CO_2_ with a technological breakthrough. In fact, most of the studies in the literature show that DAC technology cost will be reduced with time. The cost of capturing CO_2_ was estimated to reach 30, 71 and 105 EUR per ton CO_2_ based on optimistic, realistic, and pessimistic scenarios by Broehm *et al.*^73^ Nemet and Brandt^74^ also estimated a low DAC cost of 45, 23 and 14 EUR per ton CO_2_ by 2029, 2050 and 2100, respectively based on the assumed 10% learning rate and a lifetime of 50 years, which is higher than other assumptions in the literature by 20 years. Mahdi *et al.*^69^ proposed that the line of research should be more intensive on low temperature solid sorbent compared to high temperature aqueous solutions because it has higher potentials for cost reduction to reach 54 EUR per ton CO_2_ for low temperature systems compared to 71 EUR per ton CO_2_ for high temperature system by 2050 based on 15% learning curve. The reason for this potential is the fact that low temperature solid sorbents can use low thermal grade heat, does not required external water and has high modularity.

In the recent years, considerable efforts have been made by carbon capture researchers to collate work on direct air capture of CO_2_ in the form of review articles. Several review articles have been published on DAC sorbent materials and technology options.^75–80^ For example, the work of Deng *et al.*,^75^ Zhu *et al.*,^76^ Cherevotan *et al.*,^77^ and Shi *et al.*^78^ focused on various forms of sorbent materials for DAC applications, while McQueen *et al.*^79^ provided analysis on current as well as future DAC technologies, and Erans *et al.*^80^ assessed DAC technologies with a focus on techno-economic and socio-political challenges. A search through the literature showed few review articles that combine all the highlighted elements in a single publication. This work, nonetheless, combines the review of DAC sorbent systems with technology options. Moreover, to the best of our knowledge, this work is the first article to review DAC integration with HVAC systems – an emerging technological option.

As a comprehensive review, the article starts by highlighting the importance of using porous material to capture CO_2_ from the atmosphere over any other process. All the absorption and adsorption-based technologies are assessed in detail with tables to allow for the comparison. A general overview on the commercialization of DAC is presented, and for the first time, a detailed review of the articles that touch on DAC-HVAC systems' integration is carried out. The work highlights the main challenges and benefits of the integration, presents mathematical models for the integration evaluation, proposes certain suitable technologies based on the conducted reviews and shows a brief technoeconomic study of HVAC-DAC unit integration to demonstrate the cost benefits therein.

## Description of DAC

The direct air capture (DAC) system setup consists of sorbents, contact area, and regeneration module. The sorbent is a liquid or solid material that attracts CO_2_ either chemically or physically. For CO_2_ capture to occur, ambient air is exposed to the sorbent material through the contact area, and upon saturation with CO_2_ or as desired, the sorbent material undergoes regeneration in which it is separated from the captured CO_2_ to get concentrated CO_2_ stream. The sorbent material should be reversible so that it can be used many times. The removal of CO_2_ from ultra-diluted air by heating, cooling, air compression or using membranes requires an extremely high-energy consumption.^78^ For example, 2.2 MJ mol^−1^ of CO_2_ of air-cooling is required to form dry ice and 7 MJ per mole of CO_2_ is required for 1 bar pressure drop through membrane separation.^81^ On the other hand, binding CO_2_ with sorbent materials consumes little or no energy, but the most energy required in the process is associated with removing high concentrated CO_2_ from the sorbents.^78^ In the literature, sorbents associated with DAC technology are classified into two, liquid^68^ and solid^82^ sorbents. Solid sorbents do not lose heat to evaporation as liquids; it has better kinetics and is more effective in preventing the loss of volatiles to the atmosphere.^83^ The liquid (absorption-based systems) and solid (adsorption-based systems) sorbents used in DAC systems are described below.

### Absorption based systems

Absorption is a technique used to remove CO_2_ from a gas stream into the bulk of liquid sorbent based on chemical or physical interactions. The absorption of CO_2_ can be classified into the absorbent types such as alkanolamines absorption,^84^ dual alkali absorption,^85^ aqueous ammonia absorption,^86^ sodium carbonate slurry absorption and chilled ammonia absorption. The absorption processes used in DAC systems are limited to the chemical sorbents with strong CO_2_ binding affinities.^78^ The following will include only the absorbents that are used in DAC systems.

#### Calcium hydroxide solution

Calcium hydroxide solution (Ca(OH)_2_) has strong binding energy to CO_2_, so a passive or agitated pool of the solution is used to capture CO_2_ by precipitating calcium carbonate (CaCO_3_).^53^ The separation of carbon dioxide (CO_2_) from CaCO_3_ required both drying and CO_2_ regeneration in a process called calcination at 700 °C or higher temperature. Finally, the Ca(OH)_2_ is reproduced by reacting the calcium oxide (CaO) with water in a process called slaking. The reactions involved in the cycle are as follow:

1 Ca(OH)_2_ + CO_2_ → CaCO_3_ + H_2_ Δ*H*° = −109 kJ mol^−1^

2 CaCO_3_ → CaO + CO_2_ Δ*H*° = 179.2 kJ mol^−1^

3 CaO + H_2_O → Ca(OH)_2_ Δ*H*° = −64.5 kJ mol^−1^

The use of Ca(OH)_2_ solution was implemented in DAC technology by Lackner in 1999. The energy consumed for the calcination process was 179.2 kJ per mole of CO_2_, which is higher than the minimum thermodynamic calcination energy of 109.4 kJ per mole of CO_2_.^81^ The main issues associated with Ca(OH)_2_ are that it consumes even more energy in drying, and it has limited concentration due to the very low solubility of calcium hydroxide in water.^81^

#### Sodium hydroxide solution

**Fig. 4:**
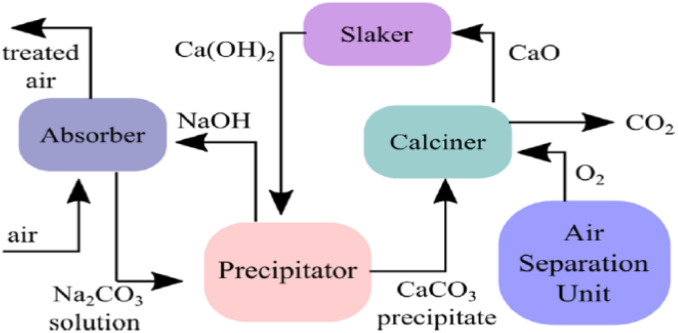
DAC based kraft process (adapted from ref. 89).

Sodium hydroxide (NaOH) has been used in the industry since 1884 in the kraft process to remove cellulose from woods. The same principle is used to capture CO_2_ and to regenerate NaOH.^87^ NaOH offers strong binding to CO_2_ like Ca(OH)_2_ with the advantage that the formed carbonate is highly soluble in water, so build-up (scaling) on the inner surfaces of the absorption column is avoided; however, the high solubility of sodium carbonate prevents direct precipitation.^88^ Separation of sodium carbonate from the solution requires high energy to evaporate a large amount of water and yield sodium carbonate. Instead, CaCO_3_ and NaOH are produced by reacting sodium carbonate with Ca(OH)_2_ in a process called causticization.^59^ The concentration of NaOH solution in causticization process is limited to 1 mole L^−1^ because at higher concentrations undesired Ca(OH)_2_ will be formed. The carbonate ions are exchanged between calcium and sodium with a theoretical efficiency of 96%.^54^ Finally, the calcination and slaking processes used in the case of Ca(OH)_2_ are also used in the kraft process to separate the CO_2_ stream and to regenerate the Ca(OH)_2_ solution as in Fig. 4 and the reactions below:

4 2NaOH + CO_2_ → Na_2_CO_3_ + H_2_O Δ*H*° = −109 kJ mol^−1^

5 Na_2_CO_3_ + Ca(OH)_2_ → 2NaOH + CaCO_3_ Δ*H*° = −5.3 kJ mol^−1^

6 CaCO_3_ → CaO + CO_2_ Δ*H*° = 179.2 kJ mol^−1^

7 CaO + H_2_O → Ca(OH)_2_ Δ*H*° = −64.5 kJ mol^−1^

From the literature, DAC based kraft process systems decompose CaCO_3_ to release CO_2_ stream at a temperature of 900 °C and the range of energy required is 1420–2250 kW h per ton of CO_2_.

#### Potassium hydroxide solution

Potassium hydroxide (KOH) is also considered an effective CO_2_ capture sorbent material that can replace NaOH using the same mentioned technique. The use of KOH was proposed as a non-toxic solution, and the results of experimental laboratory work for improved contactors were investigated.^61^ One study proposed an alternative technique in which sulfuric acid reacts with potassium carbonate to form CO_2_ and potassium sulfate (K_2_SO_4_). For completing the cycle, electrolysis with a cation exchange membrane is used to regenerate both KOH and sulfuric acid (H_2_SO_4_).^90^

#### Other alternative absorption processes

The drawbacks, such as the high-energy demand, 1 molar concentration limit, and the low efficiency associated with using Ca(OH)_2_ in the kraft process as a second absorbent inspired scientists to explore other processes in the industry. From the pulp industry, sodium metaborate was proposed to be used in the CO_2_ capture cycle instead of Ca(OH)_2_ as shown in the reactions below. It was demonstrated that borates could replace the lime in the kraft process to increase the amount of caustic available.^90^ However, the process still requires high energy and works at high temperatures, 900 °C and above.

8 NaBO_2_ + Na_2_CO_3_ → Na_3_BO_3_ + CO_2_

9 Na_3_BO_3_ + H_2_O → NaOH + NaBO_2_

Another method to directly causticize sodium carbonate without using lime is the decarbonization reaction between sodium carbonate and Na_2_O·3TiO_2_. The overall reaction enthalpy is only 90 kJ mol^−1^, which is half the energy of causticization with lime (179 kJ mol^−1^).^91^ However, the reaction requires a high temperature above 800 °C. The CO_2_ capture cycle reactions using the mentioned technique is presented below:

10 5(Na_2_O·3TiO_2_) + 7Na_2_CO_3_ → 3(4Na_2_O·5TiO_2_) + 7CO_2_ Δ*H*° = +90 kJ mol^−1^

11 Na_2_CO_3_ → Na_2_CO_3_ Δ*H*° = +25 kJ mol^−1^

12 5(Na_2_O·3TiO_2_) + 7Na_2_CO_3_ → 3(4Na_2_O·5TiO_2_) + 7CO_2_ Δ*H*° = +65 kJ mol^−1^

13 3(4Na_2_O·5TiO_2_) + 7H_2_O → 5(Na_2_O·3TiO_2_) + 14NaOH Δ*H*° = +15 kJ mol^−1^

The CO_2_ absorbed by NaOH is experimentally released using H_2_-recycling electrochemical cell. Using the cell, a pH gradient is created so that at low pH CO_2_ is released, while at high pH NaOH is regenerated. This approach regeneration energy is 374 kJ per molCO_2_.^92^ Sabatino *et al.*^93^ conducted a techno-economic study for a DAC system that uses aqueous KOH solution for absorbing CO_2_ from air while the regeneration is implemented using bipolar membrane electrodialysis (BPMED). The study showed that the regeneration energy required is 236 kJ per molCO_2_ (1491 kW h per ton CO_2_)and the total cost of capture is $773 per ton CO_2_, which is still higher energy-consuming process compared to Carbon Engineering system.^87^

**Fig. 5:**
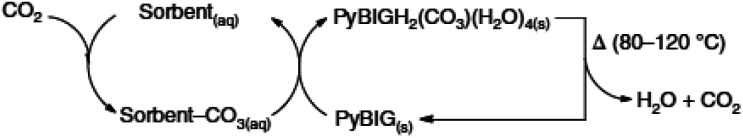
Direct air capture cycle using amino acid and guanidine compound taken from ref. 82 with permission from Springer Nature, copyright 2018.

Other absorbents are aqueous amines, which are well known materials that efficiently absorb CO_2_ from point sources with CO_2_ concentration ranging between 12–15% v/v. In the context of the binding step, aqueous solutions of NaOH or KOH are preferred over liquid amines (MEA) because NaOH or KOH solutions have faster kinetics and are stronger bases than liquid amines. NaOH has a larger capacity and can efficiently bind to a mole of CO_2_ per mole of NaOH, whereas MEA binds efficiently only to a half mole of CO_2_ per mole of MEA.^60^ In the context of regeneration, amines have a low heat of adsorption, 90 kJ per molCO_2_, compared to the traditional kraft process, 179 kJ per molCO_2_; moreover, amines can be regenerated at a low-temperature range; between 100–120 °C compared to calcination process which needs a temperature of 900 °C. The nature of capturing CO_2_ from the atmosphere (diluted source) is energy-intensive, motivating researchers to investigate different liquid amines with better CO_2_ uptake performance as amines have the potential for lower energy consumption.^94^ Hanusch *et al.*^95^ Investigated the CO_2_ uptake performance of 6 pyrrolizidine derivatives compared to MEA and linear amines. Sample number 8 (*N*-methyl-1-(tetrahydro-1*H*-pyrrolizin-7a(5*H*)-yl)methanamine) reached 90% of its capacity 3 times faster than MEA, highlighting the advantage of pyrrolizidine diamines caged structure. Moreover, the experiment showed that sample 8 achieved an absorption capacity of 1.06 molCO_2_/mole_Amine_, and it was experimentally stable for 14 cycles under pure CO_2_ absorption. For absorption from diluted air (400 ppm CO_2_), sample 6 (5-aminomethyl-1-azabicyclo[3.3.0]octane) showed no oxidation. Barzagli *et al.*^96^ screened the performance of different amines types (primary, secondary, and tertiary) and compared them to aqueous NaOH, sodium carbonate, and potassium glycinate based on a fixed concentration of 1.5 mol dm^−3^. The study shows that MEA, DGA, 1A2P, 2A1B, MMEA, EMEA, and BUMEA had CO_2_ uptake values close to NaOH and potassium glycinate based on 1 and 24 hours experiments. Other issues associated with using aqueous amines are the high energy consumed in water evaporation and high volatility and toxicity, which motivate the researchers to use organic dilutants. Vapor pressure and the higher solubility of CO_2_ in organic dilutants compared to water was reported to lead to lower regeneration energy and lower desorption temperatures.^97^ However, the aqueous amines reported capacities were higher than the same amines in organic diluents.^96^ Other liquids used to capture CO_2_ in air are aqueous amino acid solutions, which are nontoxic, nonvolatile, non-corrosive and environmentally friendly. Brethomé *et al.*^82^ reported a proof of concept that uses amino acid to absorb CO_2_ from air, and then the CO_2_-loaded amino acid reacts with guanidine compound. The result is the crystallization of a guanidinium carbonate salt, which has limited solubility and easy to be separated from the amino acid. The final step is to regenerate CO_2_ from the guanidinum carbonate salt by low thermal grade heating as shown in Fig. 5. Even with all the mentioned advantages of the indicated cycle, the equilibrium capacity and cyclic capacity of 1 M aqueous potassium glycinate solutions was reported to be only 0.76 and 0.28 molCO_2_/mol_aminoacid_, respectively. While the regeneration energy reported is 223 kJ per molCO_2,_ with a large part of that energy consumed on water evaporation. If there was no water before the CO_2_ release, the regeneration energy could be reduced to 75 kJ per molCO_2_. Cai *et al.*^98^ used trichelating iminoguanidine ligand (BTIG) to bind CO_2_ from air, forming insoluble crystal, which can be easily separated from the solution, an equilibrium capacity of 0.99 molCO_2_/mol_BTIG_ was achieved. The BTIG can be regenerated after absorbing CO_2_ by mild heating (100–150 °C), with decomposition energy consumption of 169 kJ per molCO_2_ including the heat consumed in water evaporation. It was indicated that the slow kinetics of crystallization of BTIG-CO_2_ can be two times faster by adding glycine.

#### Absorption unit limitations and novel designs

Due to the large volume of air treated by a direct air capture plant, the pressure drop is associated with a large energy penalty. Towers with filled packing materials are commonly used in the industry for absorbing a gas into solutions. The solution is dropped from the upper end of the towers while the gas is blown from the bottom. Different air contactor designs were assessed to be used for CO_2_ capture, but in the case of dilute air, large cross-section and short columns were reported to be more efficient.^59^ An example of the need for large cross sections columns to capture CO_2_ from dilute air is the study done by Keith *et al.*^59^ A capture unit in the study was designed to have an inlet and outlet CO_2_ concentration of 500 ppm and 250 ppm, respectively. The absorbent used is 1 M hydroxide sodium with a 1.44 liquid to gas ratio. The absorber was designed to allow for a pressure drop of 100 Pa m^−1^, resulting in a column with 2.8 m and 12 m in height and width, respectively.^59^ In order to reduce the pressure drop, the spray towers were proposed to be used instead of open pools and packed towers. The spray tower offers a larger surface area, reduced pressure drop, and lower construction costs compared to other designs, but also has its own energy penalty.^82^ The study^82^ also demonstrated how spray towers can reduce the air with 450 ppm CO_2_ concentration by 34 ppm, which is equivalent to absorbing 7.4 mmol L^−1^ of solution/pass. Moreover, to capture 1 ton of CO_2_, 1638 tons of atmospheric air is required, which implies large energy consumption by fans.^87^ Using the integration between DAC and natural draft from natural draft dry cooling towers (NDDCTs), the air pressure drop and fans energy penalty were avoided using the cost-free natural draft from NDDCTs instead of mechanical fans.^99^

A large portion of energy is also detected in removing moisture from calcium carbonate before the calcination process by a kiln. The average mass of water loss was reported to be 90 g of H_2_O per g of CO_2_ captured.^34^ One other limitation that increases the energy penalty is that the kiln uses oxygen instead of air to avoid CO_2_ separation from nitrogen. The whole calcination process highly consumes energy as it contributes to 4.5 GJ t^−1^ of CO_2_.^88^ In the case of spray towers, the water losses by evaporation were tested for various NaOH concentrations. The results showed that reaching a high NaOH solution concentration of 7 M could eliminate the water losses.^82^ One primary concern with spray towers is the coalescence that increases for higher flow rates and longer air contactors; however, the reduction in flow rate leads directly to a lower CO_2_ absorption rate.^82^ The proposed integration between DAC and natural draft dry cooling towers (NDDCTs) allows harvesting the consumed energy in evaporating water for improving the cooling process. The study shows that harvesting the energy increases the cooling system efficiency by 16%.^99^ The main parameters of different DAC systems based on solvents are illustrated in Table 1, and the system with the least energy consumption is associated with the use of Na_2_O·3TiO_2_ (ref. 91).

**Table 1:** DAC absorption systems specification

1st sorbent	2nd sorbent	CO_2_ ppm	Absorption *T* (°C)	Desorption^a^*T* (°C)	Reg. energy kW h t^−1^	*P*_out_ bar	Purity%	Ref.
Ca(OH)_2_^b^	None	365	Ambient	>700	—	—	—	81
CaO	None	500	365–400	800–875	—	—	>97	100
NaOH	Ca(OH)_2_	—	Ambient	900	3030^c^	100	—	59
NaOH	Ca(OH)_2_	500	Ambient	900	1678(440)	58	—	88
NaOH	Ca(OH)_2_	380	Ambient	900	1420(764)	—	—	89
NaOH	Ca(OH)_2_	—	—	900	1199–2461^d^	—	—	82
NaOH	Ca(OH)_2_	500	—	900	1695	100	—	101
NaOH	Ca(OH)_2_	—	Ambient	900	—	—	—	69
NaOH	Ca(OH)_2_	400	Ambient	—	e	—	—	99
NaOH	HRES	DAC conditions				95		92
NaOH	Na_2_O·3TiO_2_	400	5–25	860	f	15	Pure	91
KOH	Ca(OH)_2_	400	Ambient	900	1458(366)	150	97.1	87
KOH	Ca(OH)_2_	400	Ambient	900	1458(77)		>97	87
KOH	None	355	Ambient	—	1945^g^	—	—	90
KOH	BPMED				1491			93
Potassium glycinate	Guanidine	400		80–120	1409			82
BTIG		DAC conditions	100–150	1068				98

a The exact required thermal energy is considered not the primary energy, and heat recovery was considered.

b Refers to estimation based on theoretical study.

c Without any recovery.

d The values range because of different contactors.

e Using the natural draft effect instead of fans saved great amount of electrical energy besides the advantage to the cooling system.

f 50% less high-grade heat.

g The whole power consumption not only the regeneration.

### Adsorption based systems

Adsorption is a reversible process where the solid adsorbent captures the molecules, atoms or ions of gases and liquids on its surface by physical means like van der Waals forces or by forming chemical bonding. The reversibility of adsorption process is a function of temperature and pressure, which means the adsorption and desorption capability can vary by varying both temperature and pressure. CO_2_ can be adsorbed at high pressure and then released when the pressure is lowered in a process known as pressure swing adsorption (PSA). The same process could be implemented by alternating the temperature in a process called temperature swing adsorption (TSA).^16^ The solid adsorbents will be divided in the present work among the three main categories, which are physisorption, chemisorption, and moisture swing adsorption.

In chemisorption, CO_2_ binds strongly to the adsorbent by chemical bonding, which has the capacity to capture CO_2_ even in ambient air. However, it needs high energy to release the CO_2_ from the sorbents through heating like what occurs in the calcination process.^102^ On the other hand, the physisorption requires less regeneration energy but it has low CO_2_ selectivity and capacity for atmospheric concentrations.^56^ Another process that does not consume much energy in releasing CO_2_ is the electrochemical CO_2_ capture, but it has been reported that it is only effective if CO_2_ concentration falls between 15% and 30%.^102^ Finally, moisture swing adsorption combines the advantages from both chemisorption and physisorption, as it offers a high selectivity and capacity with low regeneration energy. In the moisture swing adsorption, CO_2_ can bind to the sorbent materials if it is dry, while the separation occurs if the material is wet. The energy consumption in the process is associated with water evaporation for drying the sorbent material.^103^ Different physisorption, chemisorption and moisture swing sorption materials will be discussed in the later sections.

#### Physisorption

In physisorption, CO_2_ physically binds to the surface of the sorbent materials using interaction forces such as van der Waals forces. The evaluation of effective CO_2_ adsorbent depends on selectivity, capacity, adsorption & desorption rate, stability, and adsorption & desorption temperature. Capacity and kinetics of adsorption are affected by both adsorbent chemical composition and structure, while the CO_2_ separation mainly depends on adsorption kinetics, difference in equilibrium concentration and molecular sieving mechanisms. Although physisorption requires less energy compared to chemisorption, the usage of physisorption in CO_2_ capture has capacity and selectivity limitations.^56^ There are various materials used in physisorption that can be classified based on their pore sizes (microporous, mesoporous and amorphous) or on their chemical composition such as zeolites, activated carbon and metal organic frameworks. Based on the literature,^53,56,57^ chemisorption materials are more relevant to DAC compared to physisorption materials. As a result, the present work will give a quick look at different physisorption materials that were used in DAC and their potentials and limitations.

#### Zeolites

**Fig. 6:**
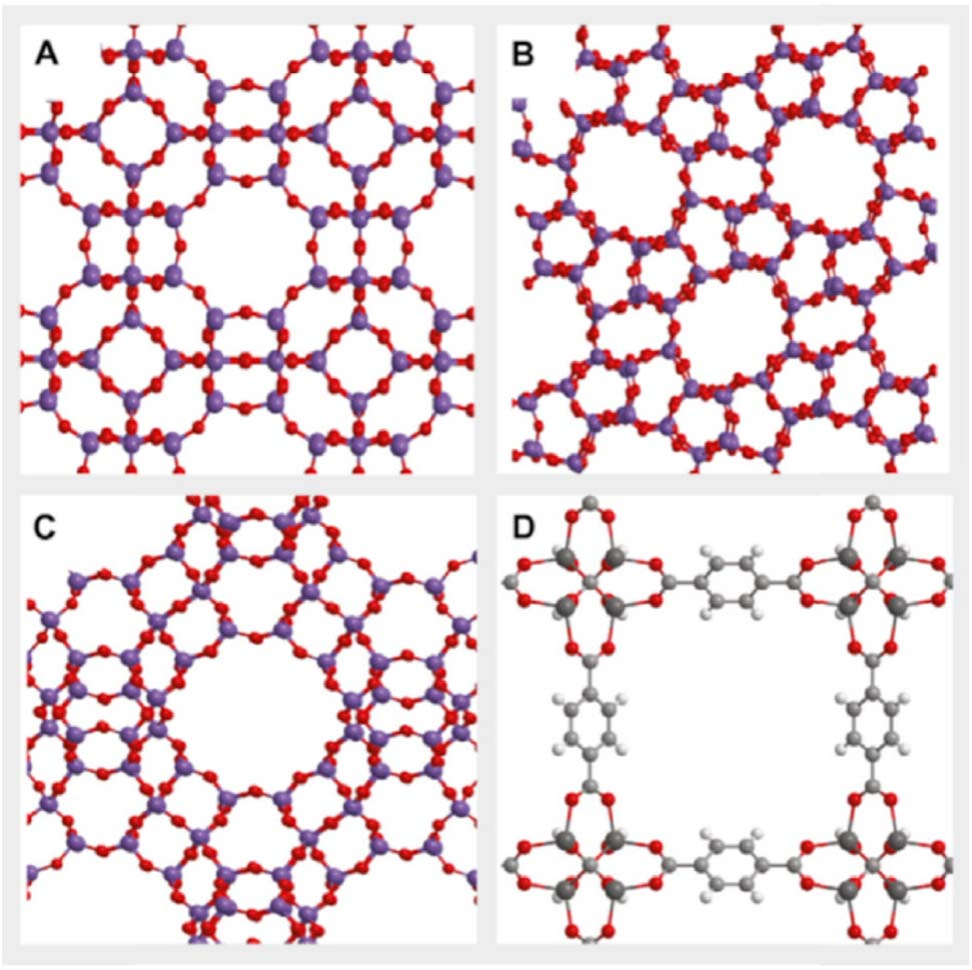
Structures of zeolites and metal organic framework (MOF): (A) zeolite A, (B) ZSM-5, (C) zeolite X and (D) MOF-5 taken from ref. 16 with permission from Elsevier, copyright 2021.

Zeolites are materials that compose of aluminum, oxygen, and silicon; they are microporous crystalline materials with matrix channel cavities and high porous surface area as shown in Fig. 6. Adsorption using zeolite is desirable in the temperature range between 0 °C and 100 °C and pressure range between 0.1 to 1 bar of CO_2_ pressures. The adsorption capacity of zeolites decreases as the temperature increases, so it is preferred in cold streams. A negative charge is induced from the silicate cations, which results in the capacity of zeolite to adsorb different flue gas components. It was reported that increasing the K^+^/(K^+^ + Na^+^) ratio in zeolites until 17% can make adsorption of CO_2_ high compared to a negligible adsorption of N_2_.^104^ The adsorption capacity of different commercial zeolite materials (APG-II, WE-G 592, 13X, 5A, and 4A) was compared, and it was reported that 13X has the highest adsorption capacity. The hydrophilic nature of zeolite, which causes strong water adsorption usually leads to low selectivity of CO_2_ in direct air capture amidst atmospheric air constituents. It was reported that increasing the K^+^/(K^+^ + Na^+^) ratio in zeolites until 17% can make adsorption of CO_2_ high compared to a negligible adsorption of N_2_.^104^ The adsorption capacity of different commercial zeolite materials (APG-II, WE-G 592, 13X, 5A, and 4A) was compared, and it was reported that 13X has the highest adsorption capacity. The hydrophilic nature of zeolite, which causes strong water adsorption usually leads to low selectivity of CO_2_ in direct air capture amidst atmospheric air constituents.^22^

#### MOFs

Metal organic framework (MOFs) are organic and inorganic materials with crystalline pores. These materials compose of organic molecules that surrounds positive metal ions. MOFs have large surface area, low density, and porosities up to 6000 m^3^ g^−1^, which make MOFs a potential adsorbent,^105^ however, MOFs have poor adsorption capacity for low CO_2_ partial pressure compared to zeolite and activated carbon (0.1–0.2 bar).^106^ MOFs adsorption from the ambient air requires very high CO_2_ partial pressure, moreover, adsorption selectivity is negatively affected by the presence of moisture in air.^107^ A recent study designed a DAC system using MOFs-polymer nanocomposite. The study reported CO_2_ purity between 70% and 80% with high water vapor repellence.^107^ compared to zeolite and activated carbon (0.1–0.2 bar).^106^ MOFs adsorption from the ambient air requires very high CO_2_ partial pressure, moreover, adsorption selectivity is negatively affected by the presence of moisture in air.^107^ A recent study designed a DAC system using MOFs-polymer nanocomposite. The study reported CO_2_ purity between 70% and 80% with high water vapor repellence.^107^

#### Activated carbon

**Fig. 7:**
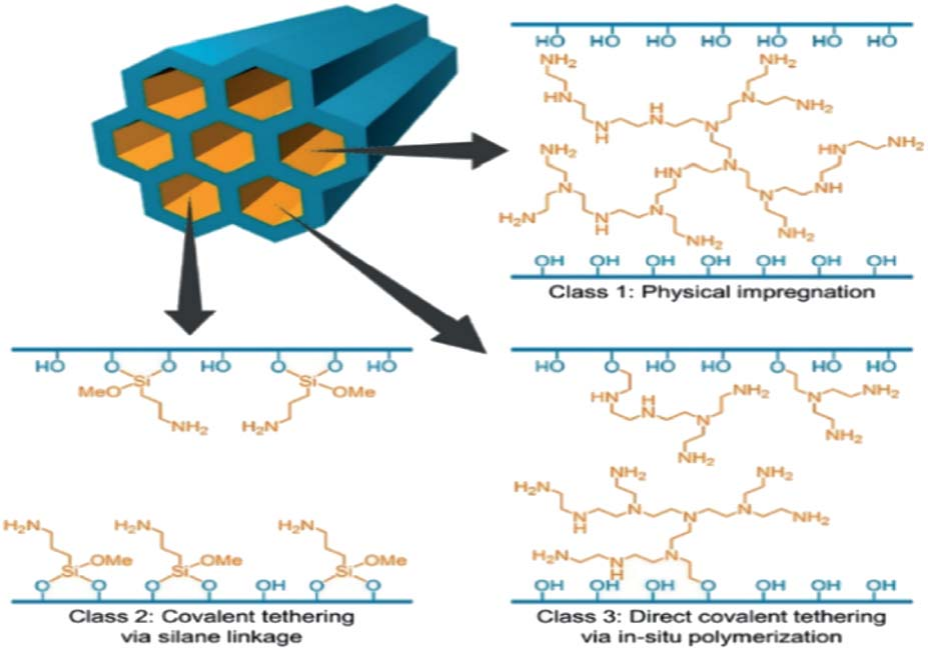
Amine modified sorbent three categories taken from ref. 78 with permission from John Wiley and Sons, copyright 2020.

Activated carbon is a charcoal that is purified and powdered then chemically or physically treated to create micro fissures that increase adsorptive surface area. It's adsorptivity is effective because of the large surface area (500–1500 m^2^ g^−1^) and electrical charge. Activated carbon is prepared from two steps, which are carbonization and activation, and it is characterized by thermal stability and low cost. Activated carbon has a lower capacity and selectivity compared to zeolites for low CO_2_ partial pressure but in higher pressure, activated carbon exhibits a higher capacity. These differences in the adsorption characteristics associated with pressure changes make activated carbon work well with pressure swing adsorption (PSA). Activated carbon selectivity towards CO_2_ in dilute air is low because of the existing moisture, but researchers are investigating different ways to improve its capacity and selectivity, but researchers are investigating different ways to improve its capacity and selectivity (Fig. 7).^78^

#### DAC relevancy summary

Although the physisorption materials consume less energy in regeneration compared to chemisorption materials, these materials have low CO_2_ capacity and selectivity in the case of atmospheric CO_2_ levels because of the existing moisture. Table 2 shows different physisorption materials that are used in DAC systems with focus on the selectivity of CO_2_ compared to water vapor. Four different physisorption sorbent materials were studied using temperature programmed desorption (TPD) and the results show that these materials have poor selectivity towards CO_2_ while the chemisorption material (TEPA-SBA-15) show much higher selectivity towards CO_2_.^108^ Other 10 physisorption materials were tested using the same technique and the results are shown in Table 2.^109^ Although the required energy in physisorption is low, the assessment of different physisorption materials show that the materials have very low selectivity towards CO_2_ compared to water vapor, which make their usage in DAC system unnecessary.^108,109^ Zeolite 13X was proposed for capturing CO_2_ from air, yet the humidity and high temperature negatively affected the zeolites. Only MOF-polymer nanocomposite shows a high selectivity towards CO_2_ as it can produce a stream with CO_2_ purity of 70–80% in presence of water vapor.^107^ The use of boron nitride nanotube and nanosheets was also proposed to be a strong CO_2_ adsorbent with high selectivity and reversibility. The materials simply adsorb CO_2_ by introducing electrons to the adsorbent and then release CO_2_ by removing the electrons.^109,110^ Although the required energy in physisorption is low, the assessment of different physisorption materials show that the materials have very low selectivity towards CO_2_ compared to water vapor, which make their usage in DAC system unnecessary.^108,109^ Zeolite 13X was proposed for capturing CO_2_ from air, yet the humidity and high temperature negatively affected the zeolites. Only MOF-polymer nanocomposite shows a high selectivity towards CO_2_ as it can produce a stream with CO_2_ purity of 70–80% in presence of water vapor.^107^ The use of boron nitride nanotube and nanosheets was also proposed to be a strong CO_2_ adsorbent with high selectivity and reversibility. The materials simply adsorb CO_2_ by introducing electrons to the adsorbent and then release CO_2_ by removing the electrons, which make them strong candidates to be used in DAC system.^111^

**Table 2:** DAC adsorption specifications using physisorption materials at 1 atm and 49% RH

Sorbent	CO_2_ con. ppm	Adsorption		Desorption		Reg. energy	TPD (mass)^a^		Ref.
		*T* (°C)	*P* (bar)	*T* (°C)	*P* (bar)	Thermal kW h t^−1^	CO_2_	H_2_O	
SIFSIX-3-Ni	∼400	∼30	—	∼140	—	—	<8% (8)	>92% (93)	108
HKUST-1	∼400	∼30	—	∼140	—	—	1%	99%	108
Mg-MOF-74	∼400	∼30	—	∼180	—	—	<4%	>96%	108
Zeolite 13X	∼400	∼30	—	∼250	—	—	1% (1.5)	99% (146)	108
SIFSIX-3-Cu	∼400	∼30	—	∼100	—	—	13.8%	86%	109
DICRO-3-Ni-	∼400	∼30	—	150	—	—	>2%	<97%	109
SIFSIX-2-Cu-i	∼400	∼30	—	∼100	—	—	1%	99%	109
MOOFOUR-1-Ni	∼400	∼30	—	∼100	—	—	>5%	<95%	109
Ni-4-PyC	∼400	∼30	—	∼100	—	—	2%	<98%	109
DMOF-1	∼400	∼30	—	∼100	—	—	2%	<98%	109
ZIF-8	∼400	∼30	—	∼100	—	—	23%	77%	109
MIL-101	∼400	∼30	—	∼100	—	—	<1%	>99%	109
UiO-66	∼400	∼30	—	∼100	—	—	<1%	>99%	108
UiO-66-NH2	∼400	∼30	—	∼100	—	—	<2%	>98%	108
MOF-polymer nanocomposite	∼400	∼15	1	80	0.1	1600	70–80%^b^		107
TEPA-SBA-15^c^	∼400	∼30	—	150	—	—	93%	7%	112

a Mass of analyte in mg g^−1^ in parenthesis.

b Produced CO_2_ purity.

c A chemosorption material for comparison.

#### Chemisorption

Chemisorption materials include both aqueous solutions and amine-modified sorbents. The aqueous solutions were discussed in the previous sections and their main drawbacks are the high regeneration energy due to their high heat capacities, and the heat required for evaporation. On the other hand, amine-modified sorbents offer a strong chemical bond and low regeneration energy, in which amines are infused into the pores of the solid materials.^110^ The adsorption of CO_2_ using amines groups occurs in two different mechanisms based on whether the condition is dry or wet. The primary and secondary amines adsorb CO_2_ to produce carbamate acid with strong bond and carbamic with weak bond, respectively in dry conditions.^113^ Chemisorption materials include both aqueous solutions and amine-modified sorbents. The aqueous solutions were discussed in the previous sections and their main drawbacks are the high regeneration energy due to their high heat capacities, and the heat required for evaporation. On the other hand, amine-modified sorbents offer a strong chemical bond and low regeneration energy, in which amines are infused into the pores of the solid materials.^110^ The adsorption of CO_2_ using amines groups occurs in two different mechanisms based on whether the condition is dry or wet. The primary and secondary amines adsorb CO_2_ to produce carbamate acid with strong bond and carbamic with weak bond, respectively in dry conditions.^113,114^ The second mechanism in wet condition, the reaction between amines and carbon dioxide forms bicarbonate.^115^ The reaction between tertiary amines and carbon dioxide to produce carbamate does not occur because there should be a hydrogen atom that can be replaced by COO^−^. The reactions between secondary amines and CO_2_ for both dry and wet conditions are shown, respectively.^115^ The reaction between tertiary amines and carbon dioxide to produce carbamate does not occur because there should be a hydrogen atom that can be replaced by COO^−^. The reactions between secondary amines and CO_2_ for both dry and wet conditions are shown, respectively.

14 2R^1^R^2^NH + CO_2_ ⇌ 2R^1^R^2^NH_2_^+^ + 2R^1^R^2^NCOO^−^ ⇌ 2R^1^R^2^NH + 2R^1^R^2^NCOOH

15 R^1^R^2^NH + CO_2_ + H_2_O ⇌ (R^1^R^2^NH_2_^+^)(HCO_3_^−^)

There are different amine-modified sorbents, which can be divided into three preparation-based categories such as category 1, category 2 and category 3 as shown in ref ^57^. All the sorbents that involve physical impregnation of amines into porous materials is included in category 1.^116–119^

Category 2 depends on stabilizing the sorbent by chemically grafting the amine to the surface of the sorbent.^120^ In category 3, the prepared amine containing monomers through *in situ* polymerization is chemically grafted to a sorbent with inorganic supports. One of DAC's startup companies, Global Thermostat, has patented different amine-modified sorbent for its operation.^78^

#### Category 1

**Fig. 8:**
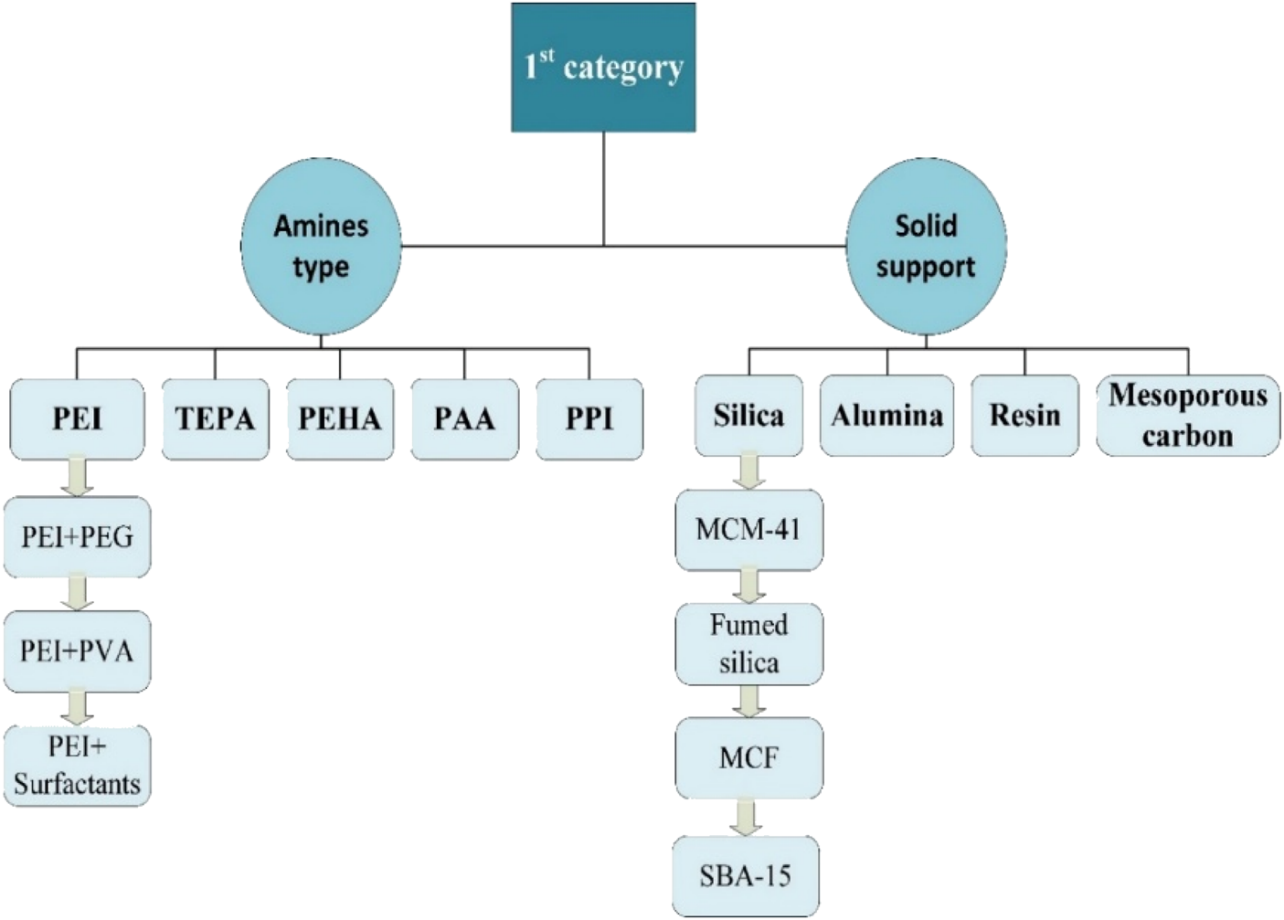
Different types of amines and solid sorbent in the first category.

The first category includes impregnating amines on solid supports to selectively capture CO_2_. There are different types of amines and solid supports reported in the literature as shown in Fig. 8. Low molecular weight and low volatility amines are preferred as this increases the capacity and stability of the sorbents. Small amines, which are used in solution separation such as monoethanolamine are not considered because of their low boiling point.^121^ Other linear amines such as pentaethylenehexamine (PEHA) and tetraethylenepentamine (TEPA) have been proposed but they have leaching and amine loss issues. The primary amine used by many researchers is branched poly(ethylenimine) (PEI), which is characterized by high density and stability under TSA and VSA. In general, PEIs are amines that are connected to ethylene, but have different shapes based on the type of amines.

**Fig. 9:**
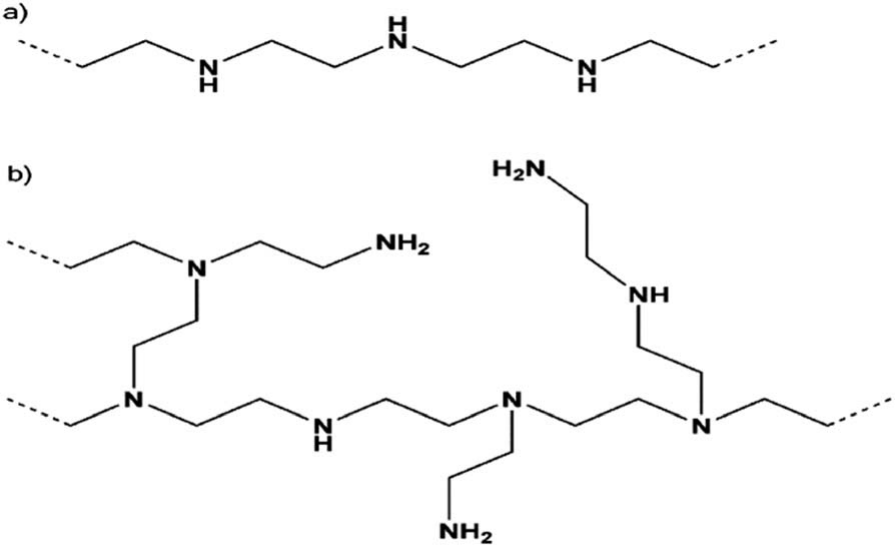
Different PEI structures (a) linear (b) branched taken from ref. 78 with permission from John Wiley and Sons, copyright 2020.

The linear shape is presented when ethylene is connected only to secondary amine while it is branched like trees when tertiary amine is included as illustrated in Fig. 9.^122^ The high heat of adsorption was expected to be an indication of higher CO_2_ capacity. The primary amines are reported to have higher heat of adsorption compared to secondary amines. The use of poly(allylamine) (PPA) that contains only primary amines has been proposed. However, a comparison between PPA and branched PEI was conducted and the branched PEI achieved higher performance in capturing CO_2_.^123^ Later, it was reported that secondary amines give the best balance between CO_2_ uptake and energy requirements.^124^ Another attempt to improve the efficiencies of amine was the addition of poly(ethylene glycol) (PEG) to PEI. The addition of PEG1000 to PEI-SBA-15 increased the CO_2_ uptake from 0.61 to 0.79 mmoleCO_2_ per g.^125^ The use of hexamethyldisilazane (HMDS) is another way to improve CO_2_ capacity of the sorbent. The small-molecule poly(propylenimine) (PPI) and PEI/PVA^126^ were proposed to be used instead of PEI to avoid oxidative degradation at high temperatures. Using PPI, the stability was simulated to last over 50 cycles without loss in performance.^127^ The internal diffusion of CO_2_ inside the PEI film can be enhanced using diffusion additives such as span 80 (sorbitan monooleate produced by Sinopharm) leading to higher CO_2_ capacity.^76^ The main solid sorbents associated with the first category are discussed below.^122^ The high heat of adsorption was expected to be an indication of higher CO_2_ capacity. The primary amines are reported to have higher heat of adsorption compared to secondary amines. The use of poly(allylamine) (PPA) that contains only primary amines has been proposed. However, a comparison between PPA and branched PEI was conducted and the branched PEI achieved higher performance in capturing CO_2_. Later, it was reported that secondary amines give the best balance between CO_2_ uptake and energy requirements.^124^ Another attempt to improve the efficiencies of amine was the addition of poly(ethylene glycol) (PEG) to PEI. The addition of PEG1000 to PEI-SBA-15 increased the CO_2_ uptake from 0.61 to 0.79 mmoleCO_2_ per g.^125^ The use of hexamethyldisilazane (HMDS) is another way to improve CO_2_ capacity of the sorbent.^128^ The small-molecule poly(propylenimine) (PPI) and PEI/PVA^126^ were proposed to be used instead of PEI to avoid oxidative degradation at high temperatures. Using PPI, the stability was simulated to last over 50 cycles without loss in performance.^127^ The internal diffusion of CO_2_ inside the PEI film can be enhanced using diffusion additives such as span 80 (sorbitan monooleate produced by Sinopharm) leading to higher CO_2_ capacity.^76^ The main solid sorbents associated with the first category are discussed below.

#### Silica support

**Fig. 10:**
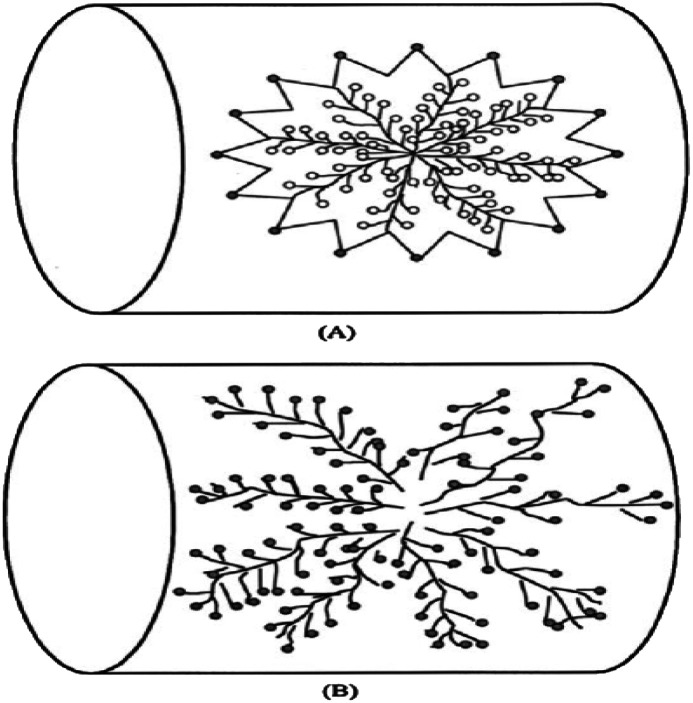
Structure of PEI in MCM-41 with ●: Active sites for CO_2_ absorption ○: hidden sites for CO_2_ absorption (a) low temperature (b) high temperature taken from ref. 122 with permission from American Chemical Society, copyright 2002.

Mesoporous silica support was first used by Xu *et al.* by adding polyethyleneimines (PEI) into the silica pores. This was the first molecular sieve with the name Mobil Composition of Matter (MCM-41). The structure of the sorbent has effect on how efficient CO_2_ binding to amines is, moreover, the structure is temperature-dependent as shown in Fig. 10.^78^ The method used to prepare the PEI in (MCM-41) is wet impregnation, where the silica porous material is added to dissolved amines in organic solvent (methanol). The main problem with the mentioned category is the instability of the polymeric amines in humid conditions due to water condensation, solubilization and their weak interaction to the solid support.^119,129,130^ The method used to prepare the PEI in (MCM-41) is wet impregnation, where the silica porous material is added to dissolved amines in organic solvent (methanol).^131^ The main problem with the mentioned category is the instability of the polymeric amines in humid conditions due to water condensation, solubilization and their weak interaction to the solid support.^119,129,130^ Moreover, the high volatility and low boiling point of monoethanolamine lead to stability issues even in dry conditions.^119^ The volatility can be avoided using large molecular weight amines, however, large molecular weight amines have been reported to have negative effects on CO_2_ capacity.^118,119,132^ The volatility can be avoided using large molecular weight amines, however, large molecular weight amines have been reported to have negative effects on CO_2_ capacity.^118,132^ Researchers have investigated the use of various PEI with different molecular weights (*M*_W_) between 400 and 25 000 in porous silica to maximize the stability.^129^

#### Amine modified alumina

Alumina^133^ and titania^134^ were proposed to replace silica to avoid silica's shortcomings. The amine modified silica regeneration methods, such as temperature-vacuum alternation, exposure to water vapor (under 110 °C) and hot CO_2_ flow lead to instability and collapse of their CO_2_ capacity. The silica support stability is limited to below 135 °C in dry condition due to urea formation, while SBA-15, MCF and MCM-41 were reported to have low stability in humid conditions.^135^ Alumina support crystallinity nature offers a high resistance to degradation when it is regenerated with water vapor. PEI modified mesoporous γ-alumina (SynA) regeneration using steam was compared to SBA-15 under the same condition. The CO_2_ capacity of SynA was reduced by 25.2% while the reduction was 81.3% in the case of SBA-15.^133^ Other amine modified solid supports such as resin,^87^ carbon nanotube^87^ and mesoporous carbon are shown in Table 3.

**Table 3:** DAC studies associated with first category of chemisorption materials

Supports	PEI type	CO_2_ con. ppm	Adsorption *T* (°C)	Desorption *T* (°C)	CO_2_ purity	CO_2_ capacity mg g^−1^	Stability mg g^−1^	Ref.
Nanosilica	B^a^	—	90	130/150	—	142–156	—	136
Nanosilica	B^a^	—	70	85	Pure	147	Minimal leaching	119
Nanosilica	L^a^	—	70	85	Pure	173	Leaching of amine	119
Nanosilica	PEI	∼400	∼25, dry	∼110	—	103.8	72.6^b^	129
Nanosilica	A-PEI	∼400	∼25, dry	∼110	—	99.46	90.2^b^	129
Nanosilica	T-PEI	∼400	∼25, dry	∼110	—	96.38	95^b^	129
—	MSiNT^c^	—	75	—	—	121	—	140
—	MMSV^d^	—	90	—	—	166.8	—	141
Fumed silica	FS-PEI-33		23 °C, RH 67%			77.89	4% – 4th cycle^e^	121
Fumed silica	FS-PEI-50		23 °C, RH 67%			62.05	4% – 4th cycle^e^	121
Fumed silica	PEI-H + PEG		50 °C, dry	—	—	29.9	Stable-20 cycle	124
SBA-15	PEI	400	75 °C, dry	50–110		22.44	Stable-20 cycle	124
SBA-15	PEI + PEG200	400	30 °C, dry			34.77	—	125
Zr-SBA-15	PEI	400	25 °C, dry			37.44	4% – 4th cycle^e^	138
SBA-15	PPI	400	35 °C, dry	110		33.44	Stable-50 cycle	137
Alumina	SynA-PEI-50	400	25 °C, dry			76.5	Constant – 3rd cycle	133
γ-alumina	PEI		30 °C, RH 50%			86.25	—	139
HP20 resin	PEI	400	25 °C			99.3	2% – 5th cycle^e^	142
Mesoporous carbon	PEI	400	25 °C, humid			99	3% – 10th cycle^e^	143
Carbon nanotube	PEI	400	30 °C, dry			47	3% – 10th cycle^e^	144

a B-branched PEI; L-linear PEI and different capacities based on different adsorption temperatures, loading, molecules weights.

b CO_2_ capacity after the 4th cycle.

c Mesoporous silica nanotube.

d Mesoporous multilamellar silica vesicle.

e Capacity reduction in the indicated cycle.

#### DAC relevancy summary

Adsorption of CO_2_ in a dry stream using polyethyleneimine modified silica was comprehensively tested under different conditions, such as different adsorption–desorption cycle temperatures (30–120 °C), loading (10–55%), molecular weights (600–2500 Da) to evaluate its cyclic stability, thermal stability, adsorption capacity and kinetics. The results showed that the higher the loading of polyethyleneimine into the silica the better its cyclic stability and CO_2_ uptake, but increasing the PEI molecular weight decreases the CO_2_ uptake with no noticeable effect on cyclic stability. PEI was found to be thermally stable below 210 °C temperature, at higher temperature, PEI starts to evaporate and decompose. The temperature variation tests demonstrate different CO_2_ uptake for different PEI loading while the cyclic stability was better at higher temperatures. In general, high loading PEI modified silica show higher CO_2_ uptake for higher temperature while for low loading, low temperatures below 60 °C is preferred. From DAC point of view, 30–40% loaded PEI modified silica is suitable as it has relatively high capacity in ambient temperature.^136^ New PEI modified silica with stabilizing additives, 3-amino-propyltrimethoxysilane and tetraethyl orthotitanate, were developed to adsorb 400 ppm of CO_2_ from inert gas stream and the results showed higher thermal stability compared to the conventional PEI; a good cyclic stability in dry conditions and a high CO_2_ capacity up to 2 molCO_2_ per kg of sorbent.^129^ The PEI was integrated into other different silica supports such as fumed silica, SBA-15, and Zr-SBA-15 as shown in Table 3. The fumed silica (FS-PEI) shows superior performance in DAC system in dry and humid conditions. Its main advantage is that it has high capacity and kinetics in humid conditions.^121^ (FS-PEI) cyclic stability was reduced by 4% after the fourth cycle, so PEI-H + PEG in dry adsorption conditions was used instead of PEI and showed a stability for 20 cycles; however, its CO_2_ capacity was lower.^124^ The use of SBA as a solid support was proposed also to be used in DAC system and it shows constant stability over 20 cycles and can reach 50 cycles^137^ using PPI instead of PEIs, but the CO_2_ capacity still was low.^124,125,138^ The fumed silica (FS-PEI) shows superior performance in DAC system in dry and humid conditions. Its main advantage is that it has high capacity and kinetics in humid conditions.^121^ (FS-PEI) cyclic stability was reduced by 4% after the fourth cycle, so PEI-H + PEG in dry adsorption conditions was used instead of PEI and showed a stability for 20 cycles; however, its CO_2_ capacity was lower.^124^ The use of SBA as a solid support was proposed also to be used in DAC system and it shows constant stability over 20 cycles and can reach 50 cycles^137^ using PPI instead of PEIs, but the CO_2_ capacity still was low.^124,125,138^ However, the amine-modified silica shows high capacity and stability under dry condition. The DAC system requires high stability sorbent in humid conditions. Alumina was proposed to capture CO_2_ from ambient air as it has high resistance to structural changes in humid conditions.^133,139^ Finally, the use of the first category for developing indoor DAC system can expose humans to the danger of inhaling amines due to the support's oxidative degradation, thermal and cyclic instability or amine group's leaching and high volatility. The main DAC studies including the use of resin and mesoporous carbon are shown in Table 3.

#### Category 2

During regeneration, a more stable category is the second category where different amine groups are chemically grafted onto the solid supports. The first category of chemisorption has a higher CO_2_ capacity than the second, however, the first category suffers from low stability due to the weak physical forces between the amine groups and the supports.^78^ In the second category, the amines covalently bind to the solid supports through the active sites (such as silyl, hydroxyl, and alkyl) on their surface.^16^ The indicated category can be prepared by two methods, which are silane chemical reactions and binding to coupling agents. The silane chemical reaction involves hydrolyzation or alcoholization of siloxane silane amines groups, which bind to a hydroxy group and condense on the surface of a support, and then a single amine layer is produced.^78^ Different studies on the second category will be discussed below and arrange based on the solid supports used.

#### Silica and its derivatives support

The use of TRI-PE-MCM-41 was first proposed by Belmabkhout *et al.*^145^ to capture CO_2_ from air in humid and dry conditions. TRI-PE-MCM-41 successfully captured CO_2_ from a mixture containing N_2_, O_2_, H_2_O vapor, and CO_2_ and showed high selectivity towards CO_2_. It demonstrates higher performance in CO_2_ capture compared to physisorption materials like Zeolites and MOFs.^145^ The effect of the support structure, such as specific surface area, pore size, and pore volume, was evaluated. The studies showed that larger pore sizes and pore volume of the support (SBA-15) offer better adsorptivity properties because they allow higher amine density. The effect of steam treatment on amine-grafted silica was also investigated, and the results show that CO_2_ capacity is reduced especially at low temperatures where the reduction becomes significant. It was illustrated that commercial-grade silica (P10) has better hydrothermal stability when compared to SBA-15.^146^

#### Alumina supports

The resilience of alumina to steam treatment is high compared to silica, which makes alumina more stable. One more difference between alumina and silica is that alumina can be tuned to be either acidic or basic. 3-Aminopropyltriethoxysilane (APS) was grafted onto two alumina support types, and it was found that different amine species are formed once the alumina interacts with carbon dioxide. The reported CO_2_ capacity was between 0.15 and 0.75 mmol (CO_2_)/g(sorbent), which is lower than the reported capacity for amine grafted silica supports when it was adsorbed from N_2_ stream with CO_2_ concentration of 500 ppm.^147^

#### Nano fibrillated cellulose (NFC) & porous polymer networks (PPN)

The Nano fibrillated cellulose consists of cellulose fibrils aggregates that exist naturally and are rich in hydroxyl groups.^148^ The use of NFC shows high stability and high CO_2_ capacity in direct air capture of CO_2_.^79^ The decrease in CO_2_ capacity of NFC after 100 cycles was only 5%, and its capacity ranged from 39.6 to 93.7 mg of CO_2_ per gram of sorbent as illustrated in Table 4. Researchers have also investigated the use of porous polymer networks (PPN) as a support instead of silica. PPNs offer higher amine loading and CO_2_ capacity compared to silica because of their three-dimensional structure and high porosity. A comparison was held between (PPN-6-CH_2_DETA) and TRI-PCM-40 (silica support) at ambient conditions. The CO_2_ capacity of PPN-6-CH_2_DETA (43.1 mg g^−1^ of sorbent) was higher by 6%, and the heat of sorption was lower by 25%.^31^

**Table 4:** DAC studies associated with second category of chemisorption materials

Supporter	PEI type	CO_2_ con. ppm	Adsorption *T* (°C)	Desorption		CO_2_ capacity mg g^−1^	Stability mg g^−1^	Ref.
				*T* (°C)	*P* (bar)			
PE-MCM-41	Triamine	400	25, RH 27%	—	—	89.77	—	145
Mesoporous Silica	MCF_APS_hi	400	25		—	70.4		152
Silica gel	Trimethoxysil ane	400	25 °C, dry	90	0.15	17.6	Stable-40 cycle	153
Silica gel	Trimethoxysil ane	400	25 °C, RH 40%	90	0.15	19.36	Stable-40th cycle	153
Porous alumina	APS	400	39, dry			6.6–33	—	80
NFC	APS	400	25 °C, RH 40%	90		61.17	Stable-20th cycle	154
NFC	AEAPDMS	400–530	30 °C, RH 60%	90	0.03	39.6	5% – 100th cycle	31
NFC	APS	Dilute	23 °C, dry			48.85	Stable-20 cycle	155
NFC	APS	Dilute	25 °C, RH 91%			93.74	Stable-20 cycle	155
RFAS4	APS		30 °C, RH 4%	80		74.37	Stable-10 cycle	156
MOF	Diamine		25 °C, dry			124.54	4% – 20th cycle	149
MOF	Diamine		25 °C, dry			68.2	Stable-5 cycle	150
MOF	Diamine		25 °C, dry			171.19	Stable-5 cycle	151
MOF	Alkylamine		20 °C, dry			49.29	Stable-15 cycle	152

#### MOF supports

Ethylenediamine based metal–organic frameworks (Mg/dobpdc) was used to capture CO_2_ from ambient air, and its CO_2_ capacity was 125.4 mg of CO_2_ per gram of adsorbent,^7^ which is higher than other MOFs adsorbents.^149^ The isotherm shape and kinetics of Mg/dobpdc for CO_2_ adsorption under dilute air conditions lead to lower CO_2_ adsorption. It was found that the mentioned adsorbent did not fully saturate with CO_2_ under the low CO_2_ partial pressure in the atmosphere due to the strong molecules' bonds between the two adjacent amine groups. The authors raised the adsorbent CO_2_ capacity to 171.19 mg g^−1^ under atmospheric conditions by using diamine: hydrazine (H_2_N_4_) instead of ethylenediamine.^150^ Brønsted acid–base reactions method was used to tether alkylamines to the Cr-MIL-101-SO_3_HMOF. The developed adsorbent under the optimal conditions showed a CO_2_ capacity of 49.29 mg g^−1^ CO_2_ from the ambient air.^151^

#### DAC relevancy summary

The major issue with using the first category in DAC systems is that the material losses stability, which implies a higher cost besides the environmental concern of releasing the adsorbent material directly into the atmosphere.^78^ The second category was proposed to overcome these issues, however, a reduction of CO_2_ capacity was noticed. A comparison between different primary, secondary and tertiary amines grafted on mesoporous silica was carried out to evaluate which one is the most suited to ambient air conditions. It was found that amines with higher primary amines have higher CO_2_ capacities and stronger water affinity. Moreover, it was reported that lower adsorption temperature achieved higher CO_2_ capacity, which is relevant for DAC systems.^152^ Diamine-functionalized silica gel was used to capture CO_2_ from ambient air using the TVS method, and it shows cyclic stability up to 40 cycles, but its CO_2_ capacity was very low compared to other adsorbents.^33^ The amine-based nano fibrillated cellulose was also proposed to be used in DAC, and it showed a high cyclic stability as it decayed only 5% after 100 cycles under humid conditions, but the presence of O_2_ led to sorbent degradation.^148^ Although the adsorption of H_2_O with CO_2_ using APS based NFC was reported to increase the CO_2_ capacity, it also contributed to higher energy requirements at the regeneration stage.^79^ The limitation raised from adsorption of water, such as high regeneration energy and instability at high temperatures, motivated the integration between hydrophobic aryl moieties and alkylamines to increase the selectivity of alkylamines to CO_2_ over water. Different amines bearing benzene moieties were investigated, and among them, *m*-xylylenediamines(MXDA, 4e), *o*-xylylenediamines(OXDA, 4g) and *p*-xylylenediamines(PXDA, 4f) did not adsorb any water with the CO_2_.^80^ Although the second category offers a strong chemical bond between amines and the supports, the solid supports self-degraded after a number of cycles and volatilize with amines. The mentioned issue discourages the use of second-category materials in indoor units. The main studies parameters associated with chemisorption class 2 are shown in Table 4.

#### Category 3

Although class 2 adsorbents were reported to be stable over 100 cycles in humid conditions,^31,157^ other studies showed that the stability is reduced below category 1 level upon exposure to water.^158^ Moreover, the second category adsorbents have low CO_2_ capacity due to the low loading of amines compared to other categories. The third category involves covalently bound polymeric amines on a porous solid support, which allows for higher amine loading. These types of sorbents are called hyper-branched amino silica (HAS), and they were developed by Choi *et al.*^158^ These sorbents offer high capacity, stability, easy preparation, low cost, and excellent regeneration compared to category 2. It was reported that the CO_2_ capacity of these adsorbents increases linearly, from 8.8 to 66 mg g^−1^ of adsorbent, with more amine loading under dilute air conditions.^159^ Other examples of category 3 are functionalized SBA-15 by melamine-based dendrimers and hyper-branched amino silicas.^158^ Moreover, the second category adsorbents have low CO_2_ capacity due to the low loading of amines compared to other categories. The third category involves covalently bound polymeric amines on a porous solid support, which allows for higher amine loading. These types of sorbents are called hyper-branched amino silica (HAS), and they were developed by Choi *et al.*^158^ These sorbents offer high capacity, stability, easy preparation, low cost, and excellent regeneration compared to category 2. It was reported that the CO_2_ capacity of these adsorbents increases linearly, from 8.8 to 66 mg g^−1^ of adsorbent, with more amine loading under dilute air conditions.^159^ Other examples of category 3 are functionalized SBA-15 by melamine-based dendrimers and hyper-branched amino silicas. Poly(l-lysine) brush–mesoporous silica hybrid material was also used to capture CO_2_ from dilute air and it was reported to be stable over three cycles with CO_2_ capacity of 26.4 mg of CO_2_ per gram of adsorbent under ambient air conditions.^160^

#### Chemisorption regeneration methods

One more important parameter of CO_2_ capture beside adsorbent CO_2_ capacity is the regeneration. The allowable cost of the whole CO_2_ capture and regeneration cycle will dramatically decrease if the capacity is low which ultimately leads to unrealistic process from the economy point of view.^161^ One of the regeneration methods is the pressure swing adsorption (PSA), which is viable for post combustion capture,^162^ but PSA requires very high compression or unpractical vacuum level for it to be applicable to DAC, which makes it impractical for DAC application.^153,163^ The most often-used regeneration method for amine-based adsorbent in the laboratory scale is temperature swing adsorption.^164^ Although the TSA needs simple design, most amines degrade if desorption temperature reaches above 100 °C, moreover, it produces dilute CO_2_ stream.^165^ The desorption of CO_2_ using inert gas instead of air can successfully solve the issue of oxidative degradation; however, it increases the process cost, which is not practical for DAC systems in which cost of capture is already high. The high CO_2_ purity stream could be achieved by TSA using CO_2_ as the stripping gas, but the issue is the formation of urea, which deactivates the adsorbent^166,167^ One more important parameter of CO_2_ capture beside adsorbent CO_2_ capacity is the regeneration. The allowable cost of the whole CO_2_ capture and regeneration cycle will dramatically decrease if the capacity is low which ultimately leads to unrealistic process from the economy point of view.^161^ One of the regeneration methods is the pressure swing adsorption (PSA), which is viable for post combustion capture, but PSA requires very high compression or unpractical vacuum level for it to be applicable to DAC, which makes it impractical for DAC application.^153,163^ The most often-used regeneration method for amine-based adsorbent in the laboratory scale is temperature swing adsorption.^164^ Although the TSA needs simple design, most amines degrade if desorption temperature reaches above 100 °C, moreover, it produces dilute CO_2_ stream.^165^ The desorption of CO_2_ using inert gas instead of air can successfully solve the issue of oxidative degradation; however, it increases the process cost, which is not practical for DAC systems in which cost of capture is already high. The high CO_2_ purity stream could be achieved by TSA using CO_2_ as the stripping gas, but the issue is the formation of urea, which deactivates the adsorbent^166,167^ and reduces the adsorption cycle working capacity. Another way for achieving high purity CO_2_ stream by TSA is to use saturated steam as the stripping gas and then condense the water from the product gas,^158,168^ the main issue with using steam as the stripping gas is the leaching of amine, which significantly reduces the cyclic stability^136,140^ Another way for achieving high purity CO_2_ stream by TSA is to use saturated steam as the stripping gas and then condense the water from the product gas,^158,168^ the main issue with using steam as the stripping gas is the leaching of amine, which significantly reduces the cyclic stability.^136,140^

The combination of vacuum with TSA is used as a regeneration method called temperature-vacuum swing adsorption (TVSA), it reduces the desorption temperature to below 100 °C; however, amine leaching is still the associated drawback when steam is used as the stripping gas.^169^ TVSA is capable of producing almost 100% pure CO_2_, but the required temperature swing will be high, and the working capacity will reduce when compared to TSA and temperature concentration swing adsorption (TCSA)^153,163^ used as the stripping gas.^169^ TVSA is capable of producing almost 100% pure CO_2_, but the required temperature swing will be high, and the working capacity will reduce when compared to TSA and temperature concentration swing adsorption (TCSA).^153,163^ The reason for the mentioned changes is that TVSA required closed inlet during desorption to prevent dilution of the product gas, which means that desorption occur in high CO_2_ concentration chamber.^170^ A comparison of CO_2_ capacity of diamine-functionalized silica gel based on regeneration methods (TCSA and TVSA) used.^163^ It was found that TCSA and TVSA at desorption temperature of 90 °C and vacuum pressure of 50 mbar achieved CO_2_ capacity of 19.36 and 11.88 mg of CO_2_ per gram of sorbent, respectively. It was reported that the increase in desorption temperature could increase the working capacity, but it is limited by thermal degradation. Moreover, the working capacity of adsorbent using TVSA can be increased by the regeneration when purge gas such as inert gas or air is used. However, this is not proposed for DAC systems due to the large energy penalty required for its implementation, moreover, it does not produce pure CO_2_ stream. In the literature, a comparison between desorption rate using steam or inert gas as the stripping gas with TVSA has been reported.^169^ The adsorbent regenerability is as important as working capacity and specific energy requirement (SER), as it significantly affects the DAC system cost,^161^ however, most amine-modified stability experiments are done through few cycle numbers below 20.^167^ Most of the DAC system experiments are limited to only 10 (ref. 130) cycles, however, some studies have reached up to 20.^169,171^ The adsorbent regenerability is as important as working capacity and specific energy requirement (SER), as it significantly affects the DAC system cost,^161^ however, most amine-modified stability experiments are done through few cycle numbers below 20.^167^ Most of the DAC system experiments are limited to only 10 (ref. 130) cycles, however, some studies have reached up to 20 (ref. 171) and 100 (ref. 31) cycles. Most studies that compare the regenerability based on different regeneration methods focus on the mechanism of degradation and not the cyclic stability.^172^ Considering only 100% pure CO_2_ production in DAC systems limits regeneration methods to closed temperature vacuum swing adsorption (TVSA), steam and CO_2_ stream stripping methods. There are other applications^173^ which do not need 100% pure CO_2_ such as microalgae cultivation,^174^ greenhouses^175,176^ and microbial cultivation.^177^ The required CO_2_ separation power has been estimated to vary from 1250 kW h t^−1^ to (3471–4418) kW h t^−1^ for CO_2_ production with purity less than 5% to more than 90%, respectively.^178^ The previous estimate shows that it is highly important to choose different regeneration methods based on the targeted CO_2_ purity. There are other applications which do not need 100% pure CO_2_ such as microalgae cultivation,^174^ greenhouses^175,176^ and microbial cultivation.^177^ The required CO_2_ separation power has been estimated to vary from 1250 kW h t^−1^ to (3471-4418) kW h t^−1^ for CO_2_ production with purity less than 5% to more than 90%, respectively.^178^ The previous estimate shows that it is highly important to choose different regeneration methods based on the targeted CO_2_ purity. Different regeneration methods associated with low CO_2_ purity product such as TSA, TCSA and TVSA with air or inert gas purge flow were compared to closed TVSA without purge flow, which yields nearly 100% pure CO_2_ stream. The mentioned methods were compared based on adsorbent regenerability, working capacity (WC) and specific energy requirement (SER) for proprietary amino resin on silica support as shown in Table 5. In the study, two desorption temperatures were used, which are 60 °C and 100 °C representing 90% and 99% of the maximum working capacity, respectively. All the experiments were carried out under dry conditions. The results demonstrated that inert gas or air based 500 mbar TVSA records higher working capacity than TSA, TCSA and closed TVSA; however, the least SER was achieved by isobaric TSA and TCSA. As a conclusion, the purge gas integrated to TVSA with mild vacuum pressure offers high working capacity and reasonable specific energy requirements, which makes it more viable for low CO_2_ concentration applications.^179^ Moisture swing based chemisorption.

**Table 5:** Chemisorption regeneration methods comparison^80^

Regeneration method	Adsorption conditions	WC (mg g^−1^)		SER (kW h t^−1^)		Stability	
		60 °C	100 °C	60 °C	100 °C	WC reduction/cycle – 60 °C	WC reduction/cycle – 100 °C
TSA, air purge	400 ppm CO_2_, 25 °C	18	23.85	1250	1778		
TCSA, N_2_ purge	400 ppm CO_2_, 25 °C	19	24.24	1222	1750		0.18%
TVSA, 25 mbar, air purge	400 ppm CO_2_, 25 °C	22.35	24.82	7277	10 667		
TVSA, 500 mbar, air purge	400 ppm CO_2_, 25 °C	20.37	24.42	1916	2778		
TVCSA, 25 mbar, N_2_ purge	400 ppm CO_2_, 25 °C	22.4	24.78	7250	11 583		
TVCSA, 500 mbar, N_2_ purge	400 ppm CO_2_, 25 °C	22.66	26.36	1722	2500		
TVSA closed, T ramp	400 ppm CO_2_, 25 °C	5.37	15.18	3250	2361		
TVSA closed	400 ppm CO_2_, 25 °C	—	16.987	—	2083		0.38%
TVSA, 200 mbar	400 ppm CO_2_, 25 °C					0.26%	0.6%

#### Moisture swing mechanism

Through the moisture swing, the adsorbents bind to CO_2_ under dry condition and then the CO_2_ is released when the adsorbents are exposed to moisture. The moisture swing technology was initially proposed to be used for CO_2_ capture from ambient air by Lackner.^180^ Most of the adsorbents that use moisture swing method are quaternary ammonium ions integrated with resin, which is considered a strong basic ion exchange resin. The role of quaternary ammonium ions is similar to the role of sodium cation in aqueous solution. In carbon capture application using moisture swing, the integrated cation is balanced by hydroxide, carbonate and bicarbonate anions, which their abundance depends on the CO_2_ and moisture loading.^181^

**Fig. 11:**
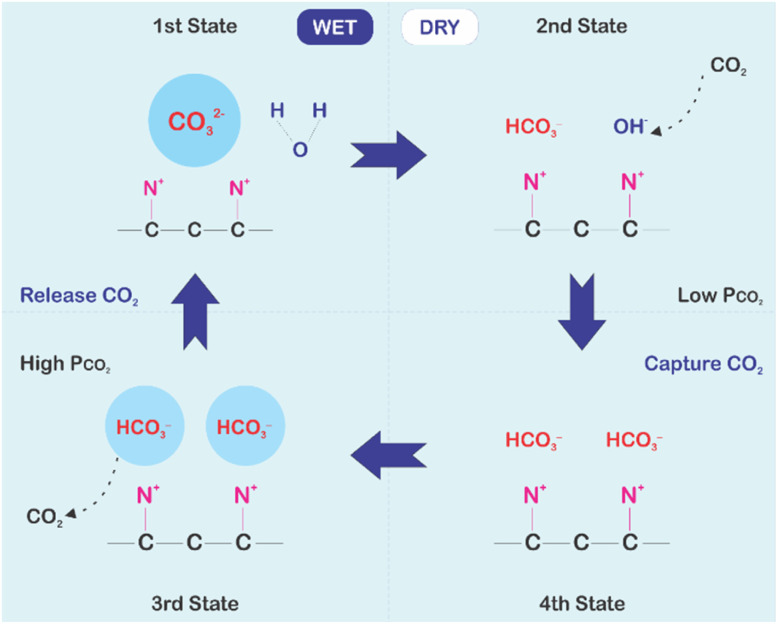
Moisture swing steps for CO_2_ capture adapted from ref. 78 with permission from John Wiley and Sons, copyright 2020.

The moisture swing mechanism can be explained based on four main steps as shown in Fig. 11. In the first step, the carbonate ions are attracted to the positive quaternary ammonium ions in the presence of water. The existence of excess water can stabilize carbonate, hydroxide or bicarbonate ions but since the carbonate hydration is stronger than the others, only the carbonate exist in the first step. In the second step, the adsorbent is dried to reduce the water content, which leads to less stable carbonate ions. The carbonate ions tend to split and react with the remaining water molecules, forming bicarbonate ions. Since the water content is reduced, there will be hydroxide ions that has high binding affinity to carbon dioxide. The reaction between both hydroxide ions and carbon dioxide leads to adsorption of CO_2_ and forming bicarbonate as in the third step. The reactions involved in the second and third steps are as follow:

16 H_2_O ⇌ H^+^ + OH^−^

17 CO_3_^2−^ + H^+^ ⇌ HCO_3_^−^

18 OH^−^ + CO_2_ ⇌ HCO_3_^−^

Moving from the third step to the fourth requires the exposure of the adsorbent to moisture, which increases the presence of bicarbonates over the existed carbonate and carbon dioxide, leading to a reaction (19) where the bicarbonates are split into carbonate ions, carbon dioxide and water. The carbon dioxide is being desorbed in this step while the presence of water and carbonate ions results in the cycle repetition. Based on the previous fourth step, the adsorption and desorption of carbon dioxide can be controlled by controlling the amount of water present.^182^

19 HCO_3_^−^ + HCO_3_^−^ ⇌ CO_3_^2−^ + CO_2_ + H_2_O

#### DAC relevancy summary

The use of ion exchange resin (IER) in DAC system was proposed since the energy required for traditional thermal swing or pressure swing adsorptions can almost be eliminated. Instead, the air moisture content changes can be used to implement the complete adsorption and desorption cycle. Most traditional DAC systems require high estimated energy for regeneration, 179,^89,91^ 135 and 80^57^ kJ per molCO_2_ for calcination process, decomposition of Na based sorbents, and amine based respectively, compared with IER which was reported to have low desorption heat of 32 kJ per molCO_2_. Moreover, it was experimentally reported that by increasing the humidity from 15.8% to a fully humidified condition, the equilibrium partial pressure of CO_2_ in IER increases by two orders of magnitude.^183^ Although the moisture swing needs relatively clean water to avoid resin contamination, and it produces CO_2_ stream with low purity, the vapor in air can be used to avoid water cleaning energy penalty, and the purity could be increased by integrating thermal swing or vacuum assisted mode to the technology. The low partial pressure of released CO_2_ stream was increased using improved binder that increased the air/sorbent contact area.^184^ The slow kinetics and low capacities of IER are considered as major disadvantages of the technology. Hou *et al.*^185^ tried to overcome this issue by preparing a moisture swing based polymeric material and the kinetics was significantly improved compared to other similar functionalized groups. Capturing CO_2_ from air using IER dispersed in polypropylene through moisture swing was investigated to determine the isothermal performance of the adsorbent.^183^ The effect of temperature and particle size on CO_2_ loading half time was studied by Wang *et al.*^184^ and the authors showed that half time decreases from 150 to 40 minutes by increasing the adsorption temperature from 0 to 30 °C. The half time of sorbents uses moisture swing technology was a major concern, which motivated researchers^186–188^ to develop new sorbents with improved sorption rates under ambient conditions. The moisture swing technique was demonstrated to be applicable with sorbents other than resin. The nanomaterials show formation of carbonate ions, which can split water into bicarbonate and hydroxide by controlling the amount of moisture.^182^ One example of these nanomaterials is Na_2_CO_3_ impregnated in activated carbon powder.^189^ A numerical and experimental investigation of moisture swing mechanism and the effect of surface hydrophobicity, pore size, and spacing of cations on CO_2_ capture efficiency was implemented.^181^ In the indicated study, five samples were tested but only two samples show sensitivity to moisture level as shown in,^89,91^ 135 and 80 kJ per molCO_2_ for calcination process, decomposition of Na based sorbents, and amine based respectively, compared with IER which was reported to have low desorption heat of 32 kJ per molCO_2_.^76^ Moreover, it was experimentally reported that by increasing the humidity from 15.8% to a fully humidified condition, the equilibrium partial pressure of CO_2_ in IER increases by two orders of magnitude.^183^ Although the moisture swing needs relatively clean water to avoid resin contamination, and it produces CO_2_ stream with low purity, the vapor in air can be used to avoid water cleaning energy penalty, and the purity could be increased by integrating thermal swing or vacuum assisted mode to the technology.^78^ The low partial pressure of released CO_2_ stream was increased using improved binder that increased the air/sorbent contact area.^184^ The slow kinetics and low capacities of IER are considered as major disadvantages of the technology. Hou *et al.*^185^ tried to overcome this issue by preparing a moisture swing based polymeric material and the kinetics was significantly improved compared to other similar functionalized groups. Capturing CO_2_ from air using IER dispersed in polypropylene through moisture swing was investigated to determine the isothermal performance of the adsorbent.^183^ The effect of temperature and particle size on CO_2_ loading half time was studied by Wang *et al.*^184^ and the authors showed that half time decreases from 150 to 40 minutes by increasing the adsorption temperature from 0 to 30 °C. The half time of sorbents uses moisture swing technology was a major concern, which motivated researchers^186,187^ to develop new sorbents with improved sorption rates under ambient conditions. The moisture swing technique was demonstrated to be applicable with sorbents other than resin. The nanomaterials show formation of carbonate ions, which can split water into bicarbonate and hydroxide by controlling the amount of moisture.^182^ One example of these nanomaterials is Na_2_CO_3_ impregnated in activated carbon powder.^189^ A numerical and experimental investigation of moisture swing mechanism and the effect of surface hydrophobicity, pore size, and spacing of cations on CO_2_ capture efficiency was implemented.^181^ In the indicated study, five samples were tested but only two samples show sensitivity to moisture level as shown in Table 6. Other Different investigated moisture swing-based DAC systems are shown in Table 6. Although the moisture swing DAC systems saves large amount of energy, it has low kinetics and low capacity compared to other methods.

**Table 6:** Moisture swing-based DAC studies

1st sorbent	CO_2_ con. ppm	Absorption	Desorption	Reg. energy electrical kW h t^−1^	Capacity mg g^−1^	Ref.
IER	400	Ambient, dried		316	—	68
IER	400	Ambient, dried		423–631		190
IER	400	25 °C, dried			36	183
Carbon black functionalized with hyperbranched polymers	400	Dried			6.16	191
IER in the carbonate form	Ambient	Room temperature, 5 °C dew temperature	Room temperature, 15 °C dew temperature		33.05	181
Nanostructured graphite (NG) with Na_2_CO_3_ into micropores	Ambient	Room temperature, 5 °C dew temperature	Room temperature, 15 °C dew temperature		28.64	181

### Other CO_2_ capture classifications

#### Nanomaterials for DAC

An efficient material for DAC systems should have a high adsorption capacity, low regeneration energy and high stability. These properties can be achieved using nanomaterials as they have chemical and thermal stability, high surface area, and accessible pore regions. Nanomaterial can be classified into three main categories: zero-dimensional nano-objects, one-dimension nano-objects, and nanostructured materials. Nanoparticles and nanofluids are included in the first category. Nanoparticles are small particles; their sizes vary from 1–100 nm and can be fabricated from different metals or core materials.^192^ Nanofluids are fluids with suspended nanoparticles, and their three-phase nature reduces the energy demand and enhances the absorption capacity of CO_2_ by increasing the mass transfer coefficient.^193^ Nanofiber and nanotubes^145^ are grouped under the one-dimension nano-objects. Nanofibers are nano-dimensional materials, but their length is larger than nanoscale. Nanofibers are fabricated from polymers such as cellulose nanofibers^194^ and metal nanofibers.^195^ Finally, nanostructured materials include nanocrystalline, nanocomposites, and nonporous materials. Nanocrystalline can be multi or single polycrystalline solids that have sizes less than 100 nm, such as functionalized BN nanosheets,^196^ cellulose nanocrystals,^189^ and metal dioxide nanocrystalline.^197^ The nanocomposites are a combination of different nano-objects with sizes less than 100 nm, and they can be in one, two, or three dimensions.^198^ The last in the category of nanostructured materials are nonporous materials with a framework having pore sizes less than 100 nm.^199^ In Table 7, many nano-based CO_2_ capture systems show high capacity and stability over a few cycles; however, all the experiments were not carried out at atmospheric CO_2_ concentration or in the presence of moisture. Including the ultra-dilute concentration levels and moisture contents can dramatically decrease the capacity and stability. Poly HIPE/nano-TiO_2_/PEI-50 was tested in the presence of both water and nitrogen and showed a high capacity and stability, but the tested concentration levels were not to the same as the atmospheric one, which makes poly HIPE/nano-TiO_2_/PEI-50 a potential sorbent that needs further investigations.^200^ CNF-Ph(1 : 1.5) was equally investigated, and the results showed a high capacity under DAC conditions; moreover, it was indicated that the increase in humidity further increased the CO_2_ capacity.^75^ Yanhao Deng *et al.*^75^ reviewed the performance of nanomaterials in DAC conditions, and it was concluded that the best performance achieved by cellulose nanofibrils under low concentrations, but suffered from lack of stability. On the other hand, nanosheets and nanostructured materials showed high stability, although they are not effective in capturing CO_2_ from ambient air.^201^

**Table 7:** Most recent experiments under conditions close to DAC systems using nanomaterial

sorbent	CO_2_ con. ppm	Adsorption	Capacity mg g^−1^	Stability%	Nanomaterial category	Ref.
50%PEI/SBA-15	400 ppm CO_2_/N_2_	25 °C	57.2	Robust over 10 cycles	0D	201
CNF-x-a-CNC	10% CO_2_/N_2_	30 °C	92.8	—	1D	202
CNF-Ph(1 : 1.5)	500 ppm CO_2_	25 °C, 1 bar	282.9	—	1D	75
PEI@BN	2% CO_2_/He	75 °C	137.3	Loss 6.3% after 10 cycles	2D	203
20wt% MgO-RHA	10% CO_2_/N_2_	—	200.7	Loss 7.68% in 10 cycles	Nano structured material	197
MgO/C-550	15% CO_2_/N_2_	27 °C	210	—	Nano structured material	198
Poly HIPE/nano-TiO_2_/PEI-50	CO_2_/H_2_O/N_2_ (1 : 1 : 8)	75 °C	246.5	Loss 9% in 50 cycles	Nano structured material	200

#### Green sorbents in DAC

The concept of removing CO_2_ from the atmosphere attracted many research interests around the world, but the difficulty and the general lack of applicability involved are the challenges in DAC.^203,204^ Different materials were proposed for DAC, such as metal hydroxide-based absorption and amine-supported materials.^78,164^ The metal hydroxide materials are lightweight, small in size, have large capacity, high stability, and good reliability, but they require high regeneration energy. The solid amine-based adsorption materials overcome the high regeneration energy penalty, but they have low CO_2_ capacity, and many have instability issues.^78^ All the mentioned materials are chemically prepared, implying energy and environmental impacts. As a result, green sorbents were proposed to meet the sustainability requirements. Eliminating environmental threats by using green chemistry attracted many researchers to explore green sorbents since the 12th Global Congress on Process Safety (GCPS).^205^ The green sorbents used for CO_2_ capture are bioregenerative materials and Ionic liquids (ILs), and they can adsorb CO_2_ and release water and oxygen. Many bioregenerative materials were used for CO_2_ capture from flue gas stream, such as mesoporous chitosan-SiO_2_ nanoparticles (NPs) with a capacity of 193.16 mg of CO_2_ per gram of sorbent^205^ and superhydrophobic PVDF/Si-R hollow fiber membrane, which shows a better performance than MEA.^206^ Eliminating environmental threats by using green chemistry attracted many researchers to explore green sorbents since the 12th Global Congress on Process Safety (GCPS).^205^ The green sorbents used for CO_2_ capture are bioregenerative materials and Ionic liquids (ILs), and they can adsorb CO_2_ and release water and oxygen. Many bioregenerative materials were used for CO_2_ capture from flue gas stream, such as mesoporous chitosan-SiO_2_ nanoparticles (NPs) with a capacity of 193.16 mg of CO_2_ per gram of sorbent^205^ and superhydrophobic PVDF/Si-R hollow fiber membrane, which shows a better performance than MEA.^206^ The outstanding characteristics of ILs make them an effective material for CO_2_ capture. Many studies have been conducted to investigate the physical properties of ILs.^206^ The use of ILs in carbon capture can be classified into three categories, which are amino acid ionic liquids,^207^ ILs-cosolvent,^202^ and ILs-supported materials.^206^

The amino acid ionic liquids (AAILs) are green sorbents that can replace the aqueous amine solution, and they show a higher adsorption rate compared to pure amino acids, amino acid salts, or the derivatives of ethanolamine.^207^ Different cations were integrated with AAILs such as chlorine, imidazole cations, phosphonium, and amines to be used in carbon capture.^208–210^ The highest adsorption capacity among tested AAILs was recorded for [P4442] [Suc] and [P4442]2[IDA]. Among different five investigated synthesized APC-ILs, [P4442]2 [IDA] showed the highest CO_2_ capacity of 130 mg g^−1^, and it was unchanged over five cycles under adsorption condition of 40 °C and CO_2_ partial pressure of 1 bar, however, reducing the partial pressure below 0.1 bar (still higher than CO_2_ atmospheric partial pressure) decreased the capacity to 38.46 mg g^−1^.^207^ [P4442] [Suc] showed a higher performance at a low concentration of CO_2_ compared to [P4442]2 [IDA]. It achieved approximately 220 mg g^−1^ adsorption capacity and stability over 16 cycles under adsorption conditions of 20 °C and partial pressure of 1 bar. The adsorption capacity of [P4442] [Suc] was reported to increase as the adsorption temperature reduced.^211^ Although these materials demonstrated high CO_2_ capacity and were shown to be stable, experiments under real atmospheric conditions and in the presence of moisture are required. There are two main problems associated with the application of AAIL in CO_2_ capture: the limited capacity under low CO_2_ concentration and high viscosity. Different ammonium based AAILs successfully achieved lower viscosity.^205,212^ The amino acid ionic liquids (AAILs) are green sorbents that can replace the aqueous amine solution, and they show a higher adsorption rate compared to pure amino acids, amino acid salts, or the derivatives of ethanolamine.^207^ Different cations were integrated with AAILs such as chlorine, imidazole cations, phosphonium, and amines to be used in carbon capture.^208–210^ The highest adsorption capacity among tested AAILs was recorded for [P4442] [Suc] and [P4442]2[IDA]. Among different five investigated synthesized APC-ILs, [P4442]2 [IDA] showed the highest CO_2_ capacity of 130 mg g^−1^, and it was unchanged over five cycles under adsorption condition of 40 °C and CO_2_ partial pressure of 1 bar, however, reducing the partial pressure below 0.1 bar (still higher than CO_2_ atmospheric partial pressure) decreased the capacity to 38.46 mg g^−1^.^207^ [P4442] [Suc] showed a higher performance at a low concentration of CO_2_ compared to [P4442]2 [IDA]. It achieved approximately 220 mg g^−1^ adsorption capacity and stability over 16 cycles under adsorption conditions of 20 °C and partial pressure of 1 bar. The adsorption capacity of [P4442] [Suc] was reported to increase as the adsorption temperature reduced.^211^ Although these materials demonstrated high CO_2_ capacity and were shown to be stable, experiments under real atmospheric conditions and in the presence of moisture are required. There are two main problems associated with the application of AAIL in CO_2_ capture: the limited capacity under low CO_2_ concentration and high viscosity. Different ammonium based AAILs successfully achieved lower viscosity,^205,212^ and the use of choline cation was reported to be an effective green sorbent in capturing CO_2_ from direct air.^213^ The main disadvantage of using choline-based AAILs is their decomposition under heating and their strong water adsorption.

The use of co-solvents such as polymer, water, alcohol, and eutectic solvent with pure ILs was proposed to decrease its high viscosity.^214^ The integration of polyethylene glycol (PEG) with ILs was investigated in different CO_2_ capture studies because of its low viscosity and cost compared to other co-solvents.^215^ The use of co-solvents such as polymer, water, alcohol, and eutectic solvent with pure ILs was proposed to decrease its high viscosity.^214^ The integration of polyethylene glycol (PEG) with ILs was investigated in different CO_2_ capture studies because of its low viscosity and cost compared to other co-solvents.^215^ However, adding the co-solvent to ionic liquid was able to decrease its viscosity, the rate of CO_2_ mass transfer was nevertheless still low. Integrating ILs on solid supports increased the adsorption rate compared to pure or co-solvent-based ILs. Many studies investigated the use of ILs on different solid sorbents such as porous silica,^216^ TiO_2_ surface,^217^ porous microsphere PMMA,^218^ supported ILs-membranes,^215^ and ILs capsules.^219^ Most of the experiments associated with the use of ILs on solid supports were conducted under flue gas stream with conditions different from those of DAC. However, the ILs based capsules were used to capture CO_2_ from a 100% humid stream with 5000 ppm of CO_2_/N_2_. The results showed high selectivity over N_2_, CO_2_ capacity of 66 mg CO_2_/g of sorbent and good stability,^217,220^ porous microsphere PMMA,^218^ supported ILs-membranes,^215^ and ILs capsules.^219^ Most of the experiments associated with the use of ILs on solid supports were conducted under flue gas stream with conditions different from those of DAC. However, the ILs based capsules were used to capture CO_2_ from a 100% humid stream with 5000 ppm of CO_2_/N_2_. The results showed high selectivity over N_2_, CO_2_ capacity of 66 mg CO_2_/g of sorbent and good stability.^220^

In summary, carbon capture field requires the use of green and sustainable sorbents. Different types of ILs show good performance in CO_2_ capture, but there are no DAC-based ILs studies in the literature to show how efficient ILs are in removing CO_2_ from dilute air. A general limitation of ILs is the complex and costly preparation methods.^204^

### Other CO_2_ sorbents

#### Supported Alkali carbonates

The replacement of hydroxide solutions by inorganic solids was studied by Steinfeld and co-workers^100^ as solid supports can increase the active alkali surface area, which improves the carbonation rates. In these sorbents, the alkali carbonates are impregnated into solid porous material. A prepared porous carbon support was loaded with MgO and CaO to capture CO_2_ and the study^219^ showed its adsorption increase with increase in moisture contents. The CO_2_ capture performance of potassium carbonate on different supports was conducted, and K_2_CO_3_/Al_2_O_3_ showed the highest CO_2_ capacity. Although K_2_CO_3_/Al_2_O_3_ has the highest capacity, it needs a high regeneration temperature of 350 °C, while K_2_CO_3_/AC and K_2_CO_3_/SG can be generated in temperatures between 100 and 200 °C.^221^ Supported Alkali carbonates DAC studies characteristics are shown in Table 8.

**Table 8:** DAC based supported alkali carbonates studies

supporter	sorbent	CO_2_ con. ppm	Adsorption *T* (°C)	Desorption *T* (°C)	Reg. energy thermal kW h t^−1^	CO_2_ capacity mg g^−1^	Stability	Ref.
Activated carbon	MgO/CaO	2000	20, humid	—	—	21	—	222
Activated carbon	K_2_CO_3_	400	20, humid	—	—	48.4	—	222
Activated carbon	K_2_CO_3_	5000	RT, humid	100–200	—	38.2	—	221
Silica aerogels	K_2_CO_3_	5000	RT, humid	100–200	—	38.2	—	221
y-Al_2_O_3_	K_2_CO_3_	Ambient	Ambient	150–300	1894, TSA	30–49	80 cycles	223
y-Al_2_O_3_	AlK5 and AlK10	400/He	45–85	250–350	TSA	34–37.8	5 cycles – 350 °C	224
Al_2_O_3_	K_2_CO_3_	5000	RT, humid	350	—	52	—	221
Y_2_O_3_	K_2_CO_3_	400	Ambient	150–250	TSA	28	20 cycles – 250 °C	225
—	K_2_CO_3_	—	Ambient	80–100	2083, TSA	—	—	69

#### Nanostructured carbon nitrides

Carbon nitride (CN) is a material linked to nitrogen by covalent bonds in a 2-D shape. The CNs are favorable material in capturing CO_2_ because of the existence of nitrogen on its surface, stability, and being green sorbents. Although carbon nitride has a nonporous nature which reduces its CO_2_ capturing capacity, it can be integrated with another porous support to improve its performance. At 23 °C and 0.93 bar, the CO_2_ capacity of melamine-based g-C3N4 nanosheets was as low as 7.9 mg g^−1^ of sorbent. The capacity was improved to 33.4 and 55.5 mg g^−1^ by incorporating BIF-20 and ZIF-8, respectively.^226^ The adsorption performance of PEI-based g-C3N4 was investigated, and a capacity of 75 and 165 mg g^−1^ was achieved at temperature of 25 °C and 100 °C, respectively.^227^ Moreover, the electronic proprieties of carbon nitride can bind and release CO_2_ by change in voltage and this method shows high selectivity to CO_2_ compared to N_2_, H_2_, and CH_4_. However, the CO_2_ capacity achieved using the previous approach is low (4.2 mg g^−1^), there are no energy barriers in binding and releasing CO_2_.^228^ Different mesoporous carbon nitride (MCN-7-100, MCN-7-130, and MCN-7-150) performance in CO_2_ adsorption was tested, and MCN-7-130 achieved a superior capacity of 594 mg g^−1^. The reported capacity was under conditions of 0 °C and 30 bar, while when the CO_2_ pressure decreased to the atmospheric pressure, the capacity dropped to 61.6 mg g^−1^.^229^ In summary, the carbon nitride sorbents experiment in the literature is limited to CO_2_ capture from flue gas, and further DAC studies are required, especially for the approach that uses only voltage change in binding and releasing CO_2_.

### Commercialization of DAC

**Fig. 12:**
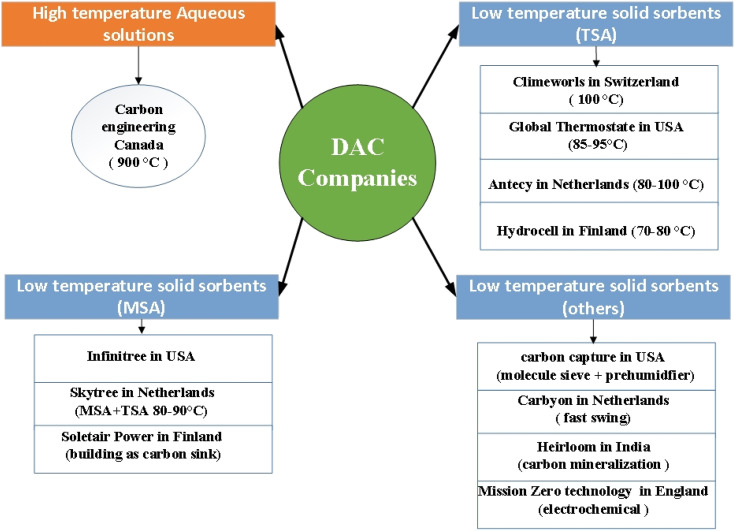
Main existing DAC companies with their regeneration methods.

Many companies work on direct air capture as shown in Fig. 12. Keith established Carbon Engineering company in Canada in 2009, in which Bill Gates partly funded it.^69^ Carbon Engineering initially used sodium hydroxide solution as a sorbent with a desorption temperature of about 900 °C. The company has recently optimized the solvent used from NaOH to KOH to enhance CO_2_ capture efficiency of their system.^230^ The company introduced a carbon capture unit that can capture 1 ton of CO_2_ per day in 2015 with a target to integrate a fuel production system based on the captured CO_2_. The company reported that it could produce 1 barrel of fuel per day using their AIR-TO-FUEL pilot in 2017. The cost of capture, purification, and compression of CO_2_ to 150 bar was estimated to be in the range of 75-113 € per tCO_2_.^63^ Another top DAC startup in the world today is Climeworks. Gebald and Wurzbacher established Climeworks company in Switzerland in 2009. Climeworks uses amine-modified sorbent to capture CO_2_ at a desorption temperature of 100 °C. Climeworks collaborated with Audi and Sunfire to launch a DAC unit that converted the captured CO_2_ into synthetic diesel in 2014.^231^ Two other commercial-scale DAC units were installed in Switzerland and Iceland to feed a greenhouse with CO_2_ and for the mineralization process, respectively in 2017. The company has a goal to achieve a cost of 75 € per tCO_2_ for the large-scale plants. In 2021, the Orca unit was launched in Iceland to capture CO_2_ from ambient air and store it through mineralization. The plant runs purely on renewable energy and can capture 4000 tons of CO_2_ per year.^232^ Eisenberger established Global Thermostat company in the USA in 2010. Global Thermostat uses a low temperature-based system to capture CO_2_ from both point sources and ambient air at the desorption temperature between 85 and 95 °C.^233^ The company launched a running unit that uses amine-modified monolith as a sorbent in California. It was reported that the waste heat with mentioned desorption temperatures could be used in a plant with a capacity of 40 000 tons of CO_2_ per year, and they target a capture cost of 11–38 € per tCO_2_.^65^ O'Connor established Antecy company in Finland in 2010. It was also working on a low-temperature sorbent that can be regenerated at a temperature between 80–100 °C^234^ later in 2019. Antecy and Climeworks companies were fully merged to achieve a better and stronger technology.^233^ Another DAC system with a CO_2_ capacity of 1.387 tCO_2_ per year uses the VSA regeneration method with regeneration temperature between 70 and 80 °C, the company was established by Oy Hydrocell.^229^ Moreover, the company provides laboratory-scale regenerative and non-regenerative CO_2_ scrubbers units.^235^ There is also Skytree company located in the Netherlands. The information about the technology used by Skytree is limited, but they use electrostatic absorption and moisture for regeneration and benzylamines functionalized sorbent.^236^ Besides Skytree, there is also Infinitree which was established in New York in 2014, and it launched a laboratory scale unit that uses moisture swing technology in capturing CO_2_.^237,238^ All other mentioned companies provide CO_2_ purity of more than 99% except companies like Skytree and Infinitree, which provide low purity CO_2_, for example, Infinitree offers CO_2_ purity between 3% and 5% for growing algae.^238^ Other newly established companies are creating innovating ideas to make direct air capture more visible such as Aircapture, Carbon Capture, Carbyon, Heirloom, Mission Zero Technologies, and Solitaire Power. Bill Gross established Carbon Capture company in California in the USA in 2019.^239^ Carbon Capture company uses molecular sieve technology to remove CO_2_ from air with a pre-dehumidifier stage powered by low-cost renewable energy. They reported that their system could produce a ton of clean distilled water with every ton of CO_2_ capture.^240,241^ Carbyon is another DAC company that was established in the Netherlands in 2019. They target a cost-effective system based on amine-modified thin membrane and fast swing technology where there is a rotating drum with a modified material to capture CO_2_ effectively. They claimed that the CO_2_ adsorption takes 30 seconds and additional 30 seconds is needed for regeneration. They currently target CO_2_ capture cost of 50 EUR per ton.^242^ Direct air capture using carbon mineralization is developed by Heirloom, a company established in South India. The carbonate minerals are decomposed into pure CO_2_ stream and oxide minerals in their system. The oxide mineral is exposed to ambient air to naturally absorb CO_2_ without any fans.^241,243^ Mission Zero technology was also launched in England in 2020 and uses electrochemical method for sorbent regeneration.^241^ Soletair Power was established in Finland in 2016, it uses buildings as carbon sinks by using resin-based DAC technology. The company proclaimed that their products boost human activity and fight climate change. They have two main products: indoor CO_2_ capture unit and building HVAC integration unit. Their products can reduce CO_2_ concentration in the airflow between 200–300 ppm and capable of adsorbing 20 tons of CO_2_ per year. However, the building HVAC integration unit is permanently fixed to the building HVAC system, whereas the indoor unit is mobile and can be moved depending on the desired location.^241,244^

## DAC and HVAC integration potential

**Fig. 13:**
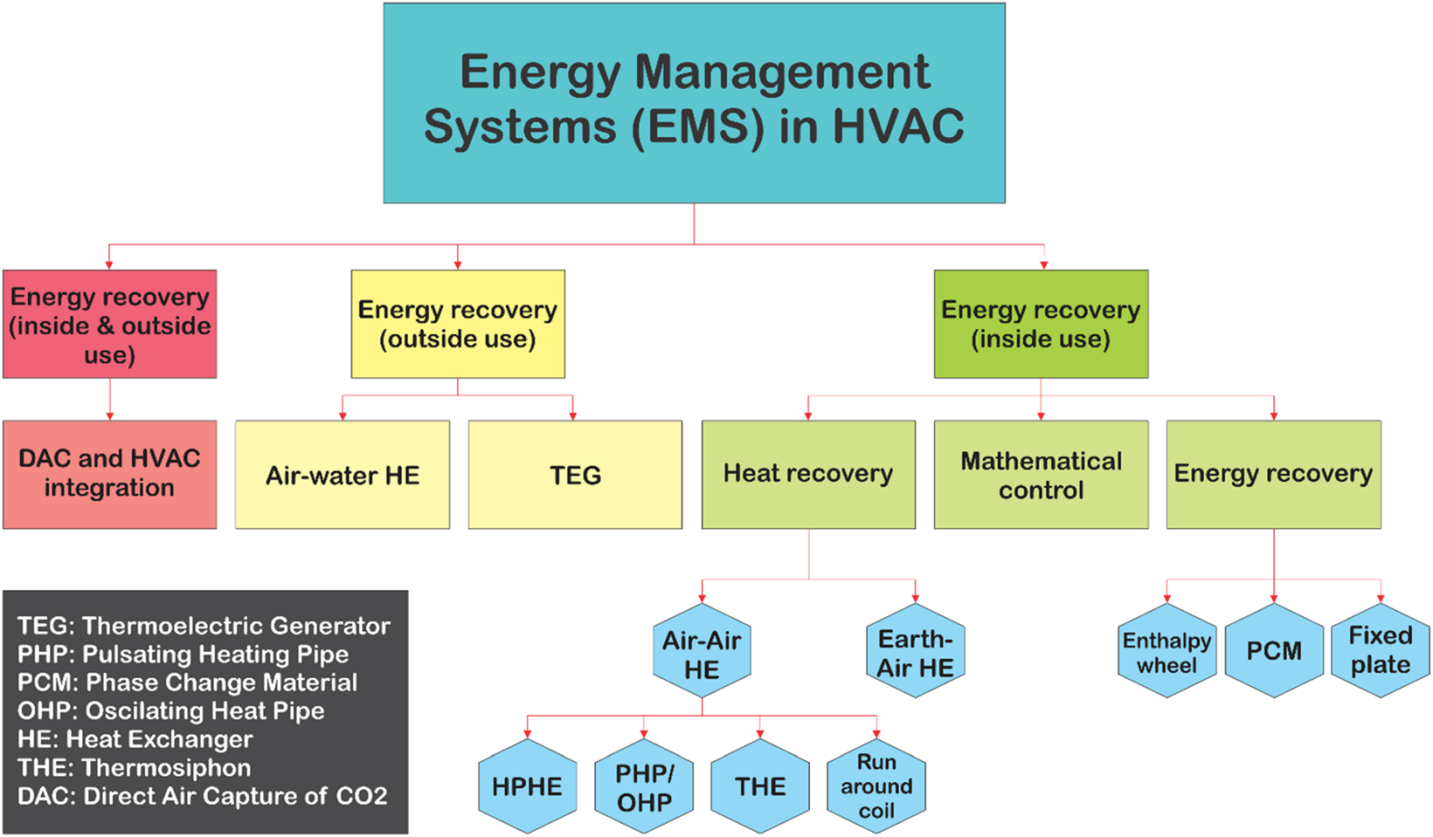
HVAC energy management classifications.

Buildings consume almost 40% of the world's energy demands, and about 50 to 82% of buildings' energy is consumed in the HVAC systems.^245^ The necessity to provide fresh indoor air, especially during and after COVID-19,^246^ and the rising energy demands, challenge researchers to reduce energy losses in the HVAC systems.^247^ Minimizing energy demands in buildings can be achieved in two ways: increasing the efficiency of the HVAC system (inside uses) or capturing the lost energy to be used for valuable purposes (outside uses).^247,248^ The building energy management systems (BEMS) were used to decrease energy consumption in HVAC systems based on the classifications shown in Fig. 13. The inside use means the recovered energy from the HVAC system is used within the HVAC system equipment to raise its efficiency in achieving thermal comfort, while the outside use means the energy is used in another system to provide a useful output such as electricity.^249^ Besides the mentioned two classifications, DAC with HVAC system integration can be proposed to combine both inside and outside uses. The inside uses include reducing indoor CO_2_ levels to improve indoor air quality (IAQ) and HVAC energy reduction using higher air recirculation ratios, while the outside uses include a more efficient DAC system by capturing from more elevated CO_2_ concentrated streams (indoor air), applying cooler adsorption and using moisture swing adsorption (MSA) within the HVAC system.

### Benefits of DAC and HVAC integration

Reducing indoor CO_2_ levels is mandatory because indoor CO_2_ accumulates due to human metabolism or emissions from indoor sources.^250^ The high levels of CO_2_ exposure lead to severe cognitive effects. Studies have shown that the CO_2_ concentrations in the range of 4000 to 10 000 cause malaise, headache and lethargy, while higher levels of 10 000 to 30 000 may cause metabolic changes, non-narcotic central nervous system and electrolyte imbalances. The indoor CO_2_ concentration can be controlled by simply replacing the indoor air with outdoor air through ventilation, however, ventilation is an energy-intensive process that adds more energy requirements for fans, heater, cooler, humidifier and dehumidifiers. Moreover, in some cases ventilation is not a solution due to the use of air exchange rate reduction strategies or in case of the need of protecting people from outdoor hazards (shelter in place). Gall *et al.*^251^ targeted reducing the CO_2_ level in indoor environment such as sleeping microenvironments, school classrooms and shelter in place (SIB) facilities as it can exceed the permissible exposure limit (PEL) of 5000 ppm in 8 hours set by Occupational Safety and Health Administration (OSHA). The study investigated four alkaline metal oxides and hydroxides Ca(OH)_2_, Mg(OH)_2_ soda lime and MgO in indoor air cleaning applications. The results showed that mg-containing sorbent has slow kinetics, then soda lime was used, and 1.7 kg was enough to reduce 80% of indoor CO_2_ levels and not exceed the OSHA PEL in SIB while it reduced the CO_2_ levels from 2599 ppm to 550–750 ppm in low ventilated bedrooms. The simulated pressure drop was 300 pa which can be achieved by typical fans from HEPA filter-containing portable air cleaner. However, CO_2_ saturated the sorbent after 40 hours based on assumed regular occupancy, implying the need for sorbent replacement, leading to high cost.

Secondly, the HVAC energy reduction can be achieved by increasing the air recirculation rate. The buildings' energy consumption is high and contributes to one-third of the total global CO_2_ emissions. However, the use of CO_2_ capture device can reduce the CO_2_ levels in building, it does not allow the full recirculation of air because of other pollutants like volatile organic compounds and dust, which are harmful to humans. Most air pollutants other than CO_2_ can be captured using filters such as HEPA H14, carbon filter, G3/4 pre-filter and F7/8 fine-filter. Although combining these filters with a CO_2_ capture unit removes all types of air pollutants,^252^ the build-up of oxygen is still an issue that requires ventilation.^253^ Based on Kim *et al.*^254^ assumption and calculations, the recirculation of air for 10 hours leads to approximately 20.12–20.174% oxygen ratio in the air, while the acceptable oxygen levels are between 19.5% and 23.5% (by volume), which allows the full recirculation of air within that period (10 hours).

A more efficient DAC system that captures from higher CO_2_ concentrated streams (indoor air) was simulated by Zhao *et al.*^255^ and the results showed that the optimal second law efficiencies for indoor CO_2_ concentration of 3000 ppm, 2000 ppm and 1000 ppm are 44.57%, 37.55% and 31.6%, respectively. The effect of Indoor CO_2_ concentration on indoor CO_2_ filters was further investigated experimentally by Hu *et al.*,^257^ the results showed that the higher the inlet concentration (1000, 1500, 2000), the steeper the breakthrough curve, which signifies a higher CO_2_ capture rate. The above studies show the potential benefits of the higher indoor CO_2_ levels on DAC unit performance. Applying cooler CO_2_ adsorption and its effects on DAC unit performance was further studied by Zhao et el.,^255^ the results showed an increase in second law efficiency from 15.38% to 39.41% when adsorption temperature changed from 323 to 298 K under 3000 ppm CO_2_ level conditions. It was explained that decreasing adsorption temperature increases the adsorbent CO_2_ capacity and reduces the minimum separation work. The previous results showed the potential for energy savings by simply locating the DAC unit in the cold region of the HVAC stream.

**Fig. 14:**
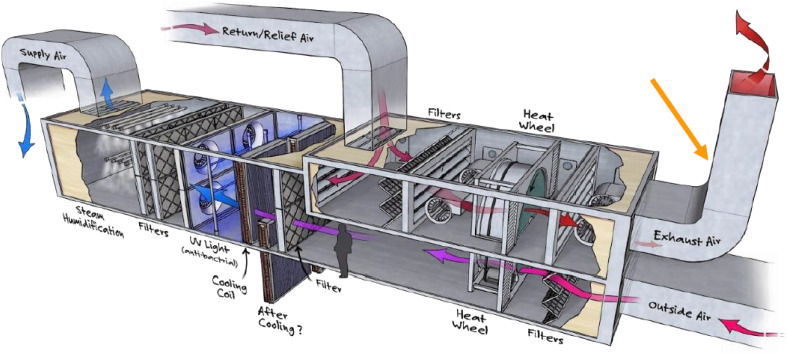
Typical Air Handling Unit (AHU) with an orange arrow indicating DAC unit position taken from ref. 256 permission from ASHRAE, copyright 2019.

Finally, the moisture swing approach in capturing and producing CO_2_ is considered the least energy consuming technique among others. However, it needs more water compared to other regeneration techniques.^258^ Shi *et al.* conducted an experiment on five different samples, showing that the higher relative humidity of air decreases the equilibrium CO_2_ concentration.^259^ The previous study highlights the potential of integrating a moisture swing-based DAC system with the HVAC system as different air streams with different humidity already exist within the HVAC system. Bryan and Salamah proposed the integration of CO_2_ collectors inside the HVAC system. The system is located to capture CO_2_ from the exhaust stream before leaving the building as shown in Fig. 14. It uses ion-exchange resin sorbent and moisture swing to adsorb and regenerate CO_2_. The advantage of their proposed system is that it uses the HVAC fan-free energy and the higher concentration of CO_2_ in the exhaust stream. Based on their calculation, the system can offset 0.1% of USA carbon emissions if the system is installed in 50% of the existing commercial buildings.^256^

### Modeling of DAC and HVAC integration benefits

**Fig. 15:**
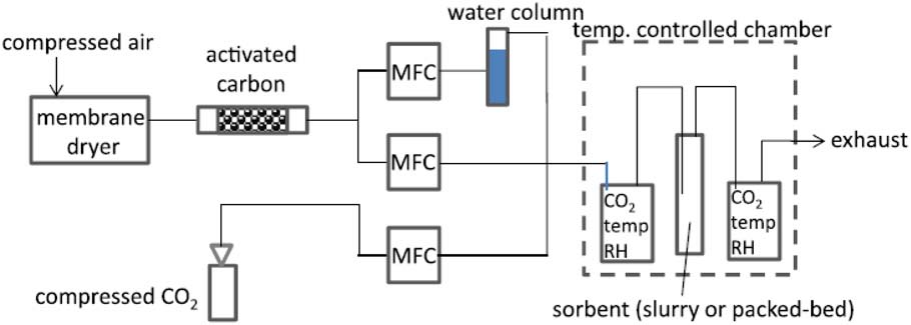
Schematic of experiment setup under controlled temperature, humidity, and CO_2_ levels taken from ref. 251 with permission from Elsevier, copyright 2016.

Different methods were used to evaluate the performance of using CO_2_ capture system within the built environment. Gall *et al.*^251^ conducted an experiment to evaluate the performance of indoor air cleaner and track the indoor CO_2_ concentration after using a CO_2_ capture unit, based on the setup shown in Fig. 15, by measuring the variation of indoor CO_2_ concentration with respect to time with and without the air cleaner. The indoor CO_2_ concentration level (*C*_room_) in mole CO_2_ m^−3^ for a room that uses a CO_2_ capture unit can be modeled by the below equation.

20 *V*_room_ d*C*_room_/d*t* = *QC*_a_ − *QC*_room_ − *Q*_f_*C*_room_ + *Q*_f_*C*_f,Last node_

where, *V*_room_ is the room volume (m^3^), *Q* is the outdoor air ventilation rate through the room (m^3^ s^−1^), *C*_a_ is the concentration of CO_2_ in outdoor air (molesCO_2_ m^−3^), *C*_room_ is the concentration of CO_2_ in the room as it enters the reactor, *Q*_f_ is the volumetric flow rate through the reactor (m^3^ s^−1^), and *E* is the total CO_2_ emission rate from occupants in the room (moles s^−1^). *Q* and *E* can be obtained based on the procedure described by Persily *et al.*,^260^ the boundary condition for the above equation is such that at *t* = 0, *C*_room_ = 0.0163 molesCO_2_ m^−3^ (or 400 ppm CO_2_) or whatever the initial concentration of room is and *C*_f,20_ (mol CO_2_ m^−3^) is the concentration of CO_2_ in the flow exiting the reactor at the last node which can be calculated based on below equations

21 *V*_f,1_·d*C*_f,1_/d*t* = *Q*_f_*C*_room_ − *Q*_f_*C*_f,1_ − *yk*[*M*_1_]*C*_f,1_*V*_f,1_

22 d[*M*_1_]/d*t* = −*k*[*M*_1_][*C*_f,1_]

23 *V*_f,*i*+1_·d*C*_f,*i*+1_/d*t* = *Q*_f_*C*_f,*i*_ − *Q*_f_*C*_f,*i*+1_ − *yk*[*M*_*i*+1_]*C*_f,*i*+1_*V*_f,*i*+1_

24 d[*M*_*i*+1_]/d*t* = −*k*[*M*_*i*+1_][*C*_f,*i*+1_]

Here, the index *i* ranges from 1 to 19, *V*_f,*i*_ is the volume of reactor element *i*, *C*_f,*i*_ is the concentration of CO_2_ in the well-mixed reactor element *i*, *M*_*i*_ is the mass of unreacted sorbent in element *i*.

To solve the above equation, parameters such as *y*(carbonation yield) and k(reaction constant) and boundary conditions need to be identified based on experimental measurements. The carbonation yield or total removed CO_2_ was calculated by measuring the CO_2_ concentration at the inlet and outlet of the indoor air cleaner and determining the time-integrated difference between both. The calculated mass of absorbed CO_2_ was divided by the initial mass of sorbent and converted to moles to determine the molar yield (*y*) in moleCO_2_/mole_sorbent_, for reaction constant (*k*) estimation, the sum of squared errors between measured and modeled values of *C*_f_ was calculated based on the following formula25
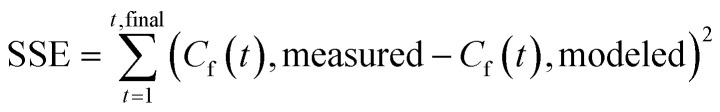


Different meaningful values of *K* were used to model *C*_f_, and then the value that achieved the minimized SSE function was used for the calculation, and finally, the boundary conditions were applied.

**Fig. 16:**
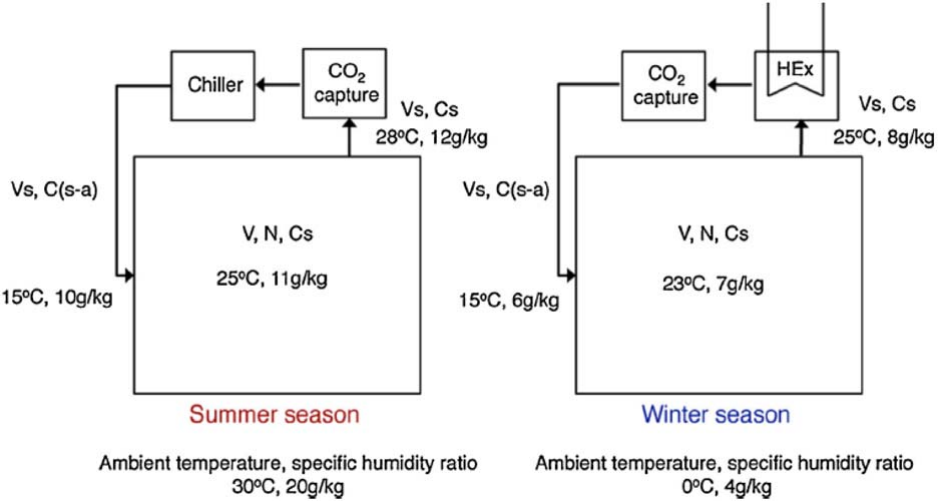
The two configurations studied by Kim *et al.*^254^ permission from Elsevier, copyright 2015.

Another study that presents a model to track CO_2_ concentration but added the energy-saving calculations by raising the air recirculation ratio was conducted by Kim *et al.*^254^ They carried out experimental and numerical modeling to analyze the effect of using a CO_2_ capture device on indoor CO_2_ concentration, allowable air recirculation ratios, and the energy-saving potentials. The new system consists of both AHU and CO_2_ capture units, which was compared to the conventional ventilation system. The modelling was implemented based on two configurations, as shown in Fig. 16.

The required outdoor airflow rate (*V*_s_) is correlated with the increase in indoor CO_2_ concentration (*C*_g_) and CO_2_ generation rate per person (*N*) with the following equation;

26 *C*_g_ = *N*/*V*_s_

While for the mechanically ventilated rooms, the equation will be;

27 *V*·d*C*/d*t* = *QC*_0_ − *QC*(*t*) + *G*(*t*)

*V* represents the room volume, *Q* is the volume flow rate, *C*_0_ is the outdoor CO_2_ concentration *C*(*t*) is the indoor CO_2_ concentration and *G*(*t*) is the CO_2_ generation rate per person, both are functions of time. The integration of the previous equation with the assumption of *Q*, *C*_0_ and *G*(*t*) to be constant lead to the following equation;

28 *C*(*t*) = *C*_0_ + *G*(*t*)/*Q* + (*C*(0) − *C*_0_ − *G*(*t*)/*Q*)e^−*It*^

29 *I* = *Q*/*V*

By adding CO_2_ capture unit to the case of air recirculation, the equation will be as follow;

30 *C*(*t*) = *C*(*t* − 1) − *A*(*t* − 1) + *G*(*t*)/*Q* + (*C*(0) − ((*t* − 1) *A*(*t* − 1))−*G*(*t*)/*Q*)e^−*It*^

where *A*(*t*) is the rate of CO_2_ adsorption. Based on the experimental work, an equation was derived with an assumption of 800 m^3^ h^−1^ air flow rate and 1 kg adsorption capacity. The equation can calculate the amount of CO_2_ adsorbed in ppm for certain time.31
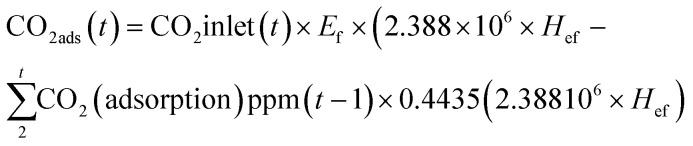
32
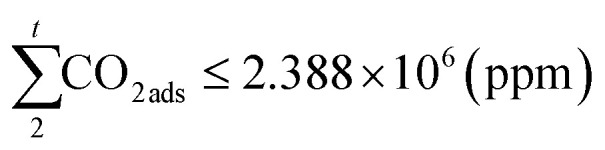


The cooling and heating loads also were calculated using the following formula;

33 *Q*_C_ = *m*_air_(*h*_in_ − *h*_out_)

34 *Q*_h_ = *m*_air_*C*_p_(*T*_in_ − *T*_out_)

**Fig. 17:**
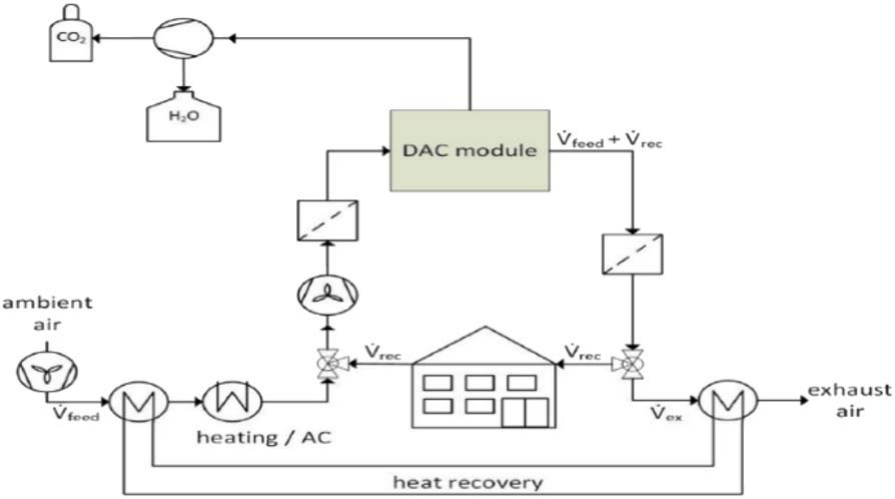
DAC and HVAC coupling scheme by Baus and Nehr^261^ with permission from Elsevier, copyright 2022.

One more model was also presented by Baus and Nehr,^261^ who investigated the possibility of attaching DAC unit to the HVAC system as shown in Fig. 17. Experimental measurements were conducted for four different buildings in Germany and the data was used in a numerical model to assess the building CO_2_ budget and potential energy saving by increasing air recirculation.

Based on the four evaluated buildings, the following equations were used to estimate building CO_2_ load and potential energy saving to be 114.3–530.2 kgCO_2_ per day and 0.34–2.57 MW h per ton.35
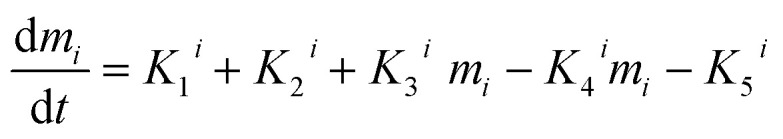

36 *Q̇*_AC_ = pair,hum·*V̇*_feed_·*C*_p,amb_·Δ*T* + 1.2*Q*_v_·ρH_2_O·*V̇*_feed_

Where *m*_i_ is the mass of either CO_2_ or H_2_O in (g), *t*_i_ is the time in (h), *K*^*i*^_1_ is the emission rate due to occupancy (g h^−1^), *K*^*i*^_2_ is the rate increasing due to ambient air feed (g h^−1^), *K*^*i*^_3_ is the rate change from recycled air, *K*^*i*^_4_ is air exchange rate in (1/h), *K*^*i*^_5_ is the performed rate of CO_2_ capture or dehumidification, *Q̇*_AC_ is the energy consumption in HVAC, *p*_air,hum_ is the specific density of humid air, *V̇*_feed_ is the ambient air rate (m^3^ h^−1^), *C*_p,amb_ is the specific heat of ambient air, Δ*T* is the temperature difference between the design point and the ambient temperature, *Q*_v_ is water enthalpy of vaporization and *ρ*_H_2_O_ is the absolute humidity of air.

The effect of both CO_2_ concentration level and cooler adsorption was modeled by a thermodynamic model presented by Zhao *et al.*^255^ The model describes the performance of TVSA based indoor CO_2_ capture unit and can be used as a method of comparison between different capturing technologies. It combines the thermodynamic second law of efficiency calculation, adsorption isotherms, and regeneration energy. The model can assess the effect of range of parameters such as adsorption temperature (*T*_ads_), desorption temperature (*T*_des_), desorption pressure (*P*_des_), indoor CO_2_ level (feed gas CO_2_ concentration *x*_A,1_), CO_2_ recovery and produced CO_2_ purity on DAC thermodynamic efficiency (*η*_TVSA_). The calculation can be performed using the following equations;37
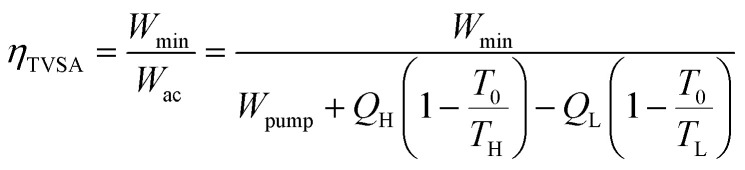
where *T*_H_ is the temperature of heat source, *T*_L_ is the temperature of cooling source, *T*_0_ is the ambient temperature and *W*_min_ is the minimum separation work, which can be calculated as follow;38
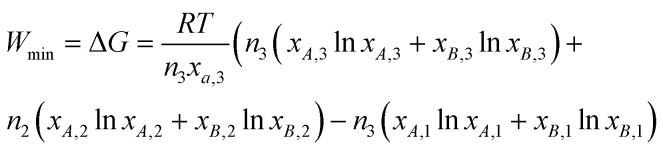
where *W*_ac_ is the actual work done which, is divided into the work done for pressurization *W*_pump_, input heat per cycle *Q*_H_ and adsorption cooling energy per cycle *Q*_L_. *Q*_H_ and *Q*_L_ can be calculated for TVSA cycle using the below formulas based on assumed single gas adsorption (CO_2_) by treating other components as inert gases, equilibrium capacity is reached, uniform temperature and CO_2_ concentration in the sorbent are attained. All other above parameters have been fully defined by zhao *et al.*^255^39
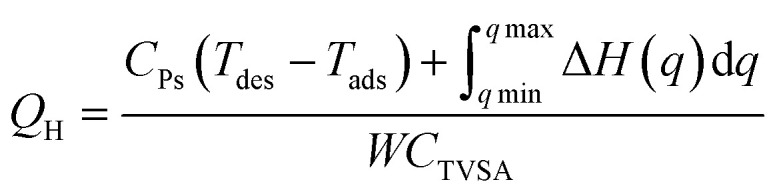
40
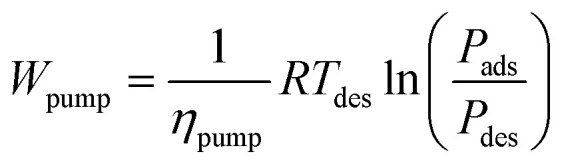

41 *WC*_TVSA_ = *q*_max_ − *q*_min_

where *q*_max_ is the maximum equilibrium CO_2_ loading in the TVSA cycle; *q*_min_ is the minimum equilibrium CO_2_ adsorption amount in the TVSA cycle, while (Δ*H*(*q*)) is the desorption enthalpy and (*q*) is the adsorption capacity, which is calculated from the isotherm curves that change from material to another. The above model used amine-functionalized cellulose and its isotherm curves parameters and calculations were obtained from ref. 163

Other models available in the literature, which are associated with filter design and adsorption kinetics were conducted by Hu *et al.*^257^ In the study, they ran an experiment to maintain acceptable IAQ levels by capturing excess CO_2_. Activated carbon filter impregnated with magnesium and calcium oxide was used as the capturing material. The filter performance was assessed based on initial removal efficiency, *η*_0_42
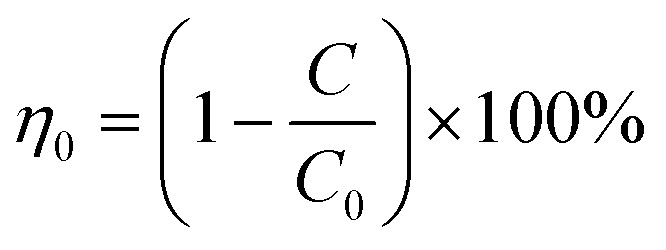
and pressure drop, which can be calculated based on Ergun equation with the interpretation of each parameter in ref. 257*et al.*43
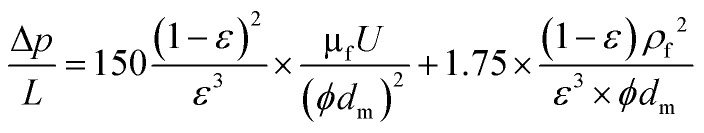


The results showed that impregnated activated carbon with MgO promotes better adsorption performance than CaO. Moreover, the experiment and model showed that the initial efficiency increases as the air velocity decreases; however, the lower air velocity leads to a higher pressure drop, indicating that both parameters should be optimized for the best performance. Lee *et al.*^262^ investigated the use of amine-modified Y-type zeolite in CO_2_ adsorption and desorption cycles under indoor environmental conditions. Three types of amines were impregnated in the zeolite (TEPA, MEA and IPA), and TEPA achieved the highest capacity of 158 mg g^−1^-adsorbent per hour under indoor CO_2_ concentration of 1000 ppm.^262^ The experimentally obtained concentrations of CO_2_ with respect to time were successfully fitted to the first-order kinetic model and it was used to calculate the rate constants *k*. The highest *k* value between the three amines was achieved by TEPA.

### Economic analysis of DAC and HVAC integration

Across different studies in the literature, it has been found that the key parameters affecting the capital and operating costs of DAC solid sorbent are the sorbent working capacity, cycle time, sorbent lifetime, desorption temperature and vacuum pressure.^79^ Many of the economic assessments performed on DAC in the literature show varied results due to differences in assumptions and methodologies; these can make it difficult for direct comparisons.^80^ The cost for current large-scale DAC systems ranges roughly between $80 per tCO_2_ to $1133 per tCO_2_, and in the future it is expected to drop to around $34 to $260 per tCO_2_.^263^ The improvements in contactor designs, sorbent properties, and heat integration are some of the parameters predicted to lower economic and environmental impacts.^263^ Bioenergy with carbon capture and sequestration (BECCS) is a competitor for DAC and its cost ranges between $20–100$ per tCO_2_ which is lower than that of DAC. Coastal blue carbon and terrestrial carbon removal are other alternatives with lower costs around $0–20 per tCO_2_. However, these technologies have drawbacks such as available land area, demand for wood and forestry management.^70^

Capital expenditures (CAPEX), operating expenditures (OPEX) and the sorbent are the main factors that affect the overall cost of DAC. The type of sorbent selected can also affect the overall cost with liquid sorbents costing less. Climeworks that uses a solid sorbent has an overall cost of $600 per tCO_2_.^160^ CAPEX contributes to most of the overall cost for this technology. Individual equipment components for the process have costs ranging from $0.13–420 million dollars.^70^ OPEX such as maintenance, labour, waste removal and makeup are necessary to keep the process running. For both liquid and solid sorbents, the OPEX is below $100 per tCO_2_ with solid systems having lower OPEX around $5 to $50 per tCO_2_.^238^ Therefore, it is necessary to conduct research to reduce costs for a more economically favourable process. Since energy is required for heating, the source of energy also influences the cost as well as the environmental impacts. Fossil fuel-based energy sources are cheaper but have a higher impact on the environment. Solar energy results in the cost being around $430 per tCO_2_, which is much higher than a fossil fuel based energy source such as coal.^264^ Since solid adsorbent systems require regeneration temperatures of around 80–120 °C, low-grade waste heat can be used.^265^

DAC coupled with HVAC can result in reduced energy requirements for ventilation systems. Additionally, when integrating with existing HVAC systems, there are available blower (fans) and existing system (duct, utilities) to use for potentially better CO_2_ capture at low temperatures. This should reduce the CAPEX for DAC as they are already available. Ji *et al.*^266^ coupled DAC with a building air conditioning system in a study, which significantly reduced the heat demand resulting in lower OPEX. They also found that not much additional cost was needed for retrofit. In a theoretical study, Baus and Nehr^261^ found that coupling DAC with HVAC in recirculation can potentially lower the energy requirements in buildings; resulting in lower operation costs. It was also highlighted that CO_2_ absorbers can be expensive and can have stability issues in the long run needing replacement. Moreover, sorbents are exposed to thermal and mechanical stress in addition to reactive chemicals, which can lower the lifetime of the sorbents. Therefore, the technical feasibility of HVAC/DAC depends on sorbents with long lifetimes and affordable costs. The economic viability is quantified by the energy saving potential of HVAC/DAC integration in recirculation mode, which lowers OPEX. Therefore, the success of the HVAC/DAC coupling depends on the DAC technology itself and the effectiveness of integration of DAC into the energy infrastructure of the building. Even though the DAC technology is in its infancy, coupling with HVAC in buildings provides an energy saving potential, which can encourage the technology to enter the mass market.^261^

#### Economic assessment methodology of DAC and HVAC integration

In this analysis, the cost assessment has been done based on a learning-by-doing model provided by Young *et al.*^267^ for a DAC system. They found that DAC combined with renewable energy can breakeven by 2030. However, with combined cycle gas turbine (CCGT) energy, they found that the system was not economically viable and needed more energy efficiency. Baus and Nehr^261^ simulated a model to assess the energy-saving potential of DAC/HVAC-coupling. They found the energy-saving potential with HVAC/DAC-coupling in recirculation mode to be between 7.85 and 52.97 MW h year^−1^ due to enhanced cooling for four different scenarios.

**Fig. 18:**
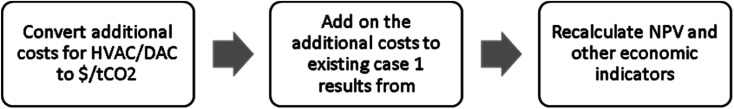
Methodology chart.

The economic model by Young *et al.*^267^ has been modified in this paper to find the economic viability of DAC/HVAC-coupling considering the energy-saving potential of cooling. The steps taken to carry out the simplistic analysis are mentioned in Fig. 18. The parameters for the economic assessment are kept the same as in the model by Young *et al.*^267^ and the costs for the energy saving are modified. The energy used in the model is provided by a combined gas cycle turbine (CCGT). The main equation used by Young *et al.*^267^ for the assessment is:

44 *c* = *c*_d_ + *c*_O&M_ = *c*_d_ + *c*_a_ + *c*_e_ + *c*_o_ + *c*_p_

where *c*_d_ is plant construction cost, CO&M is the operation and maintenance cost. The operations and maintenance consists of the absorbent/adsorbent cost *c*_a_ and the cost of energy *c*_e_ and other operations/maintenance *c*_o_, and the cost of CO_2_ processing is *c*_p_. Table 9 shows some of the input parameters used by Young *et al.*^267^ The same parameters will be considered for this study and the energy requirements will be modified. Moreover, several economic indicators such as the net present value (NPV), internal rate of return (IRR) and payback period are calculated.

**Table 9:** Input parameters for Economic assessment (data from ref. 260)

Parameters	Value
Initial cost	$394.5 per tCO_2_
Initial cost learning rate	10%
Initial absorbent cost	$37.3 per tCO_2_
Absorbent cost learning rate	5%
Initial energy demand	1535 kW h per tCO_2_
Energy demand learning rate	5%
Other O&M costs	$5 per tCO_2_
CO_2_ processing costs	25% of O&M costs

The net present value (NPV) is calculated by:45
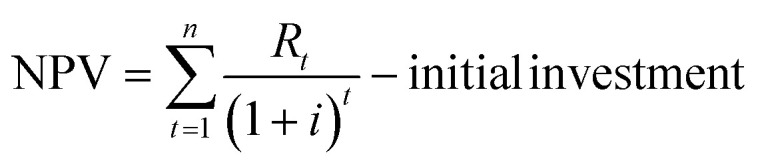
where *R*_*t*_ is the net present value, *i* is the discount rate, *t* is the time of cash flow and *n* is the time period.

The internal rate of return (IRR) is calculated by:46
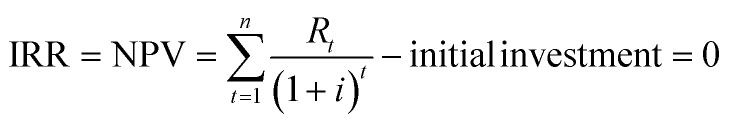
where *R*_*t*_ is the net present value, *i* is the discount rate and *t* is the time of cash flow and *n* is the time period.

The following assumptions are undertaken to conduct the economic analysis:

• All costs for the plant construction, operation and maintenance are kept the same as in Young *et al.*^267^ except the costs for energy requirement in the operations.

• It is assumed that the retrofitting costs are included in the existing model Young *et al.*^267^ in the plant construction cost.

• The CO_2_ captured is to be sold at the carbon price of $40 per tCO_2_ in 2020, peaking at $200 per tCO_2_ in 2030 and dropping to 120$ per tCO_2_ in 2050 to make the calculation simpler. Assuming linear changes between the prices.

• 52.97 MW h year^−1^ energy was saved by DAC/HVAC coupling in comparison to regular DAC^261^

• Discount rate of 7% is used for the NPV.

• All CO_2_ captured is sold.

The input parameters used in the economic modelling are given in Table 9.

#### Economic results and discussion for DAC and HVAC integration

**Fig. 19:**
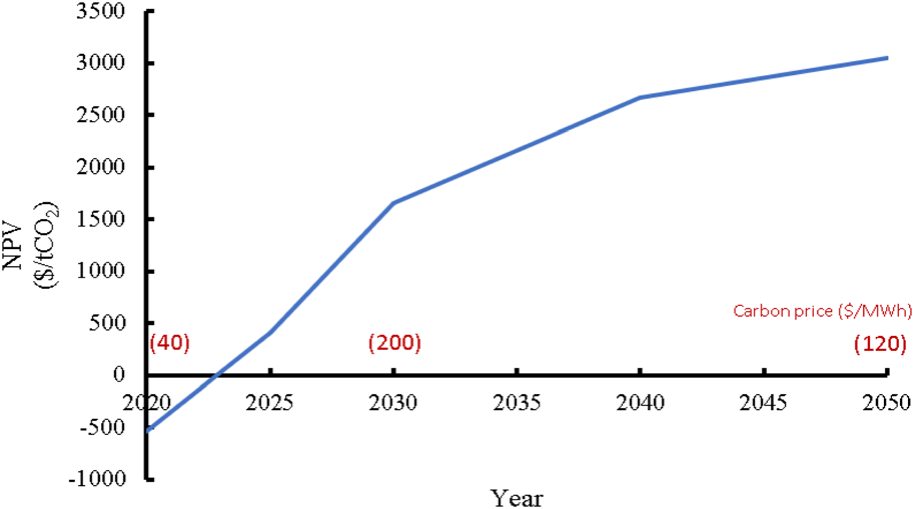
NPV over time for HVAC/DAC coupling.

**Fig. 20:**
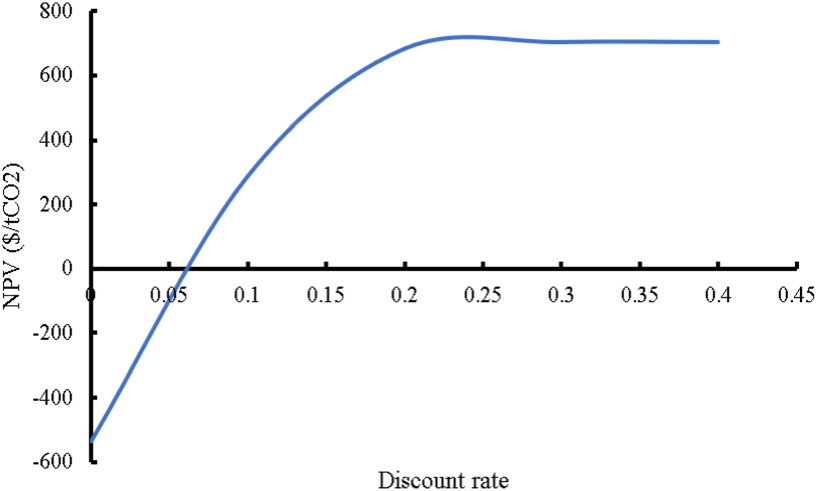
NPV *vs.* discount rate HVAC/DAC coupling.

The NPV was found to be $3047 per tCO_2_ after 50 years when the energy saving potential was assumed to be the highest at 52.97 MW h year^−1^. Fig. 19 shows that the project would reach payback period in around 3 years from the start at the point where the line crosses the origin. After that, the graph shows a positive slope showing favorable economic conditions in the future. Moreover Fig. 20 shows a graph of NPV *vs.* incremental discount rate showing the IRR to be around 0.06 (6%). Young *et al.*^267^ shows similar positive results for their cases 2–4.

**Fig. 21:**
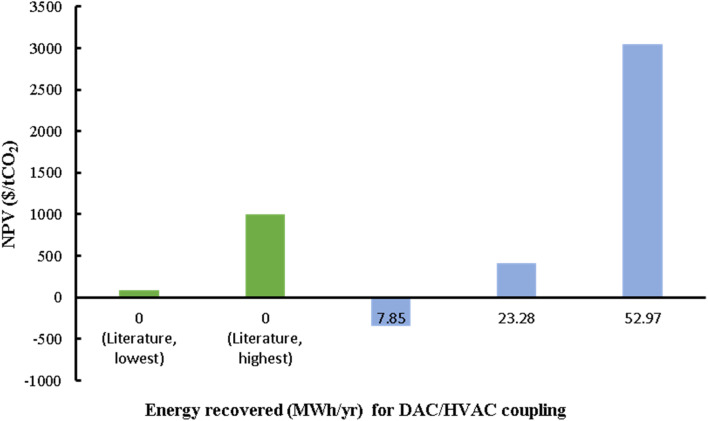
Effects of recovered energy on NPV in 50 years for this study and in literature.^268^

Energy-saving potential of 52.97 MW h year^−1^ by Baus and Nehr^261^ was for a less efficient AC; therefore, for a more modern AC unit, the energy-saving potential might be less. Hence, Fig. 21 shows how the energy-saving potential of DAC/HVAC-coupling can affect the NPV. For lower energy saving potentials, the project would not be viable using CCGT energy and renewable sources of energy would need to be used as also suggested by Young *et al.*^267^ Overall, lower energy costs and higher carbon costs would make the HVAC/DAC-coupling unit more economically favorable. As the cost of renewable energy reduces in the future, incorporating solar, wind, and geothermal technologies with DAC/HVAC could lead to a sustainable and cost-effective solution to reducing CO_2_ levels in the atmosphere. Daniel *et al.*^268^ performed a techno-economic analysis on DAC carbon capture. The study found the NPV after 50 years to range from 2 billion $ to around 24 billion$. This equates to around 82–1000 $ per tCO_2_ and the highest NPV value is shown as a comparison with this study in green in Fig. 21. The NPV for 52.98 MW h year^−1^ energy saved in this study is higher than the literature; however, with lower energy recovery in the HVAC/DAC unit the results are comparable. Therefore, coupling DAC with HVAC can be economically favorable in comparison to only DAC. Additionally, HVAC and DAC coupling have other advantages such as improved air quality due to CO_2_ reduction, energy savings due to increased air circulation and increased efficiency.

## Challenges and future recommendation in DAC-HVAC integration

The HVAC and DAC integration brings complications as well as potential savings. These complications apply to some challenges that can be explained in the following points.

• For energy efficiency purposes, full air recirculation is recommended, but the build-up of VOC requires additional filters, which increases the pressure drop in the air supply stream.

• The integration of DAC unit with the HVAC systems implies additional pressure drop in the supply air stream, which requires CO_2_ filter/sorbent material geometry's optimization.

• For the CO_2_ filter to achieve building negative emission concept, the effluent air CO_2_ level should be lower than the ambient CO_2_ level, as a result, the minimum acceptable indoor CO_2_ level should be investigated.

• The filter location should be positioned in the coldest stream; however, the coldest stream would have the highest relative humidity. The humid air will cause water adsorption and brings more energy consumption for water regeneration. This discussion raises a research challenge to find material that co-adsorb the least water molecules.

• It is important for future research in DAC-HVAC integration to utilize the cold stream for adsorbent cooling after temperature-dependent regeneration.

• For recommendations, HVAC indoor environment requires the use of environmentally friendly materials (sorbents) in order to safeguard the health of the occupants.

• Finally, investigating a way to combine humidity swings in HVAC and moisture swing adsorption of CO_2_ without mixing the produced CO_2_ with air again is recommended.

## Conclusions

This work has reviewed the literature on emerging trends in direct air capture of CO_2_. The discussion is directed at the main drivers (sorbent systems and regeneration options) of DAC for commercialization. It is well established that a good sorbent should have high CO_2_ selectivity, capacity, free binding energy, low regeneration energy, thermal stability, and cyclic stability. The two main capture options in DAC, the liquid and solid sorbents, have been tested for these characteristics. In general, the liquid sorbents are volatile, which implies heat loss due to evaporation and have lower kinetics compared to solid sorbents. For the aqueous solution of metal hydroxides, they need high temperature (up to 900 °C) for regeneration, although amine solutions have been reported with lower regeneration temperature. For solid sorbents, especially the physisorption materials, the major issue that affects their performance is their strong affinity for moisture in the atmosphere. If the strong water affinity issue is resolved, MOFs nanocomposite and porous frameworks are good sorbent candidates as they have high selectivity towards CO_2_ over N_2_ and other gases in the atmosphere. For chemisorption materials, depending on the category, the major issue affecting their performance is poor cyclic stability, which leads to leaching of amines during operations, and the poor cyclic stability is compounded when water is co-adsorbed. Although covalently bound polymeric amines on solid supports that are known as hyper-branched amino silica (HAS) have been able to solve this issue as reported. HAS offer high capacity, stability, easy preparation, low cost, and excellent regeneration compared to other categories of chemisorption materials.

As for the energy requirements for DAC system if high purity CO_2_ is desired, it should be noted that there is no significant difference in the required energy between absorption and adsorption systems. More precisely, the thermal and electrical energy requirements for solid sorbent are 3–6 GJ tCO_2_^−1^ and 1.5 GJ tCO_2_^−1^, respectively. While for liquid sorbent, the thermal and electrical energy requirements are 5.25–8.1 GJ tCO_2_^−1^ and 1.3–1.8 GJ tCO_2_^−1^, respectively, depending on the contactor configuration and packing materials. However, moisture swing adsorption (MSA) offers opportunities if high purity CO_2_ is not desired, because it has low energy consumption compared to other methods. The mechanism of MSA opens up potentials for integrating DAC with other systems such as HVAC for creating DAC system with minimum energy requirements. As illustrated in the economic analysis of DAC-HVAC integration, research efforts are already looking promising in DAC-HVAC integration; however, health impact of the sorbents to be used in HVAC systems needs to be investigated because of the sensitivity of the HVAC systems to human wellbeing. Investigation of the suitable sorbents, regeneration method, filter position, and filter design is required to gain the most of the proposed integration.

## Conflicts of interest

On behalf of all authors, the corresponding author states that there exists no conflict of interest either financially or through other personal considerations that may compromise or have the appearance of compromising the researchers' professional judgment in conducting or reporting this study.

## Nomenclature

(a-CNCs): Acetylated cellulose nanocrystals
(MCM-41): Mobil composition of matter
°C: Degree celsius
AAILs: Amino acid ionic liquids
AC: Activated carbon
APC-ILs: Aminopolycarboxylate-based ILs
APS: Aminopropyltriethoxysilane
BIF-20: A zeolite with high density of exposed B–H bonding
BN: Boron nitride
CN: Carbon nitride
CNF: Cellulose nanofibers
Con.: Concentration
Da: Dalton
DAC: Direct air capture
EMS: Energy management system
FS: Fumed silica
g: Gram
GCPS: Global Congress on Process Safety
GHG: Green house gases
GJ: Gigajoule
HAS: Hyperbranched amino silica
HIPE: High internal phase emulsion
HMDS: Hexamethyldisilazane
HVAC: Heating, ventilation, and air conditioning
IER: Ion exchange resin
ILs: Ionic liquids
IPCC: Intergovernmental panel on climate change
K^+^: Potassium cation
kJ: Kilo joule
kW h: Kilowatt hours
L: Litre
m: Meter
m^3^: Meter cube
MCF: Mesoporous cellular foam
MCN: Mesoporous carbon nitride
MEA: Monoethanolamine
Mg: Magnesium
mg: Milligram
MJ: Megajoule
MOF: Metal–organic frameworks
Na^+^: Sodium cation
NDDCTs: Natural draft from natural draft dry cooling towers
NFC: Nano fibrillated cellulose
NG: Nanostructured graphite
NPs: Nanoparticles
NRC: Nation Research Council
Pa: Pascal
PEG: Poly(ethylene glycol)
PEHA: Pentaethylenehexamine
PEI: Poly(ethylenimine)
PMMA: Poly(methyl methacrylate)
PPA: Poly(allylamine)
PPI: Poly(propylenimine)
ppm: Parts per million
PPN: Porous polymer networks
PSA: Pressure swing adsorption
PVA: Polyvinyl alcohol
PVDF/Si-R: Polyvinylidene fluoride and superhydrophobic silica nanoparticles
R^1^NH_2_: Primary amines
R^1^R^2^NH: Secondary amines
R^1^R^2^R^3^N: Tertiary amines
Ref.: Reference
Reg.: Regeneration
RFAS4: Four letter from (resorcinol, formaldehyde, 3-aminopropyltriethoxysilane)
RHA: Rice husk ash
SBA-15: Santa barbara amorphous-15
SER: specific energy requirement
SG: Silica aerogels
SynA: Mesoporous y-alumina-supported PEI composite
t: Ton
TCSA: Temperature concentration swing adsorption
TEPA: Tetraethylenepentamine
TPD: Temperature programmed desorption
TSA: Temperature swing adsorption
TVSA: Temperature-vacuum swing adsorption
VSA: Vacuum swing adsorption
WC: Working capacity
ZIF-8: Zeolitic imidazolate framework
Zr-SBA-15: Zirconium-containing mesostructured SBA-15

## Greek letters

€: Euro
Δ*H*: Enthalpy of the reaction
IPA: Isopropanol amine
IPA: Isopropanol amine
M: Molecular weigh

## Subscripts

th: Thermal

## Chemical formulas

(CH_4_): Methane
(CO_2_): Carbon dioxide
(H_2_O): Water
(N_2_O): Nitrous oxide
(O_3_): Ozone
Ca(OH)_2_: Calcium hydroxide
CaCO_3_: Calcium carbonate
CaO: Calcium oxide
H_2_SO_4_: Sulfuric acid
He: Helium
K_2_SO_4_: Potassium sulfate
KOH: Potassium hydroxide
MgO: Magnesium oxide
N_2_: Nitrogen
Na_2_CO_3_: Sodium carbonate
NaBO_2_: Sodium metaborate
NaOH: Sodium hydroxide
SiO_2_: Silicon dioxide
TiO_2_: Titanium dioxide

## Supplementary Material
